# Isoxazole Nucleosides as Building Blocks for a Plausible Proto‐RNA

**DOI:** 10.1002/anie.202211945

**Published:** 2022-10-11

**Authors:** Felix Xu, Antony Crisp, Thea Schinkel, Romeo C. A. Dubini, Sarah Hübner, Sidney Becker, Florian Schelter, Petra Rovó, Thomas Carell

**Affiliations:** ^1^ Department of Chemistry Ludwig-Maximilians-Universität München Butenandtstr. 5–13 81377 Munich Germany; ^2^ Current address: Max Planck Institute of Molecular Physiology Otto-Hahn-Straße 11 44227 Dortmund Germany; ^3^ Current address: Institute of Science and Technology Austria (ISTA) Am Campus 1 3400 Klosterneuburg Austria

**Keywords:** Isoxazoles, Origin of Life, Prebiotic Chemistry, Proto-RNA, RNA

## Abstract

The question of how RNA, as the principal carrier of genetic information evolved is fundamentally important for our understanding of the origin of life. The RNA molecule is far too complex to have formed in one evolutionary step, suggesting that ancestral proto‐RNAs (first ancestor of RNA) may have existed, which evolved over time into the RNA of today. Here we show that isoxazole nucleosides, which are quickly formed from hydroxylamine, cyanoacetylene, urea and ribose, are plausible precursors for RNA. The isoxazole nucleoside can rearrange within an RNA‐strand to give cytidine, which leads to an increase of pairing stability. If the proto‐RNA contains a canonical seed‐nucleoside with defined stereochemistry, the seed‐nucleoside can control the configuration of the anomeric center that forms during the in‐RNA transformation. The results demonstrate that RNA could have emerged from evolutionarily primitive precursor isoxazole ribosides after strand formation.

## Graphical abstract

Prebiotically plausible isoxazole nucleosides are shown to be excellent candidates for proto‐nucleosides and proto‐RNAs. The proto‐nucleoside forms 2 instructive H‐bonds with guanosine and can directly rearrange in RNA to give cytidine. The stereochemical outcome of the reaction is controlled by already present canonical seed‐nucleosides.

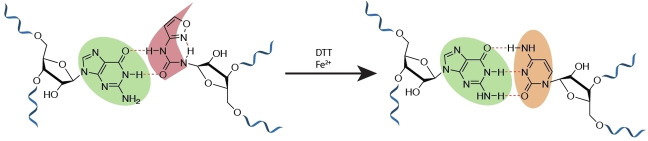

All concepts that have been developed to explain how RNA oligonucleotides could have formed on the early Earth are based on the prebiotic formation of nucleosides, which oligomerize in an enzyme free reaction.[^1^, ^2^, ^3^, ^4^, ^5^, ^6^] These concepts are based on the idea that nucleosides must have initially formed in prebiotic reactions, after which they were phosphorylated[^7^, ^8^, ^9^, ^10^] and subsequently oligomerized (Figure 1a).[^2^, ^11^, ^12^, ^13^, ^14^, ^15^] This scenario requires complex multi‐step prebiotic reactions to generate the nucleosides as a prerequisite for RNA formation.^[16]^ The complexity of this scenario has early on led to the idea that RNA emerged through a process of chemical evolution from a much simpler proto‐RNA (first ancestor of RNA) precursor molecule.[^17^, ^18^, ^19^, ^20^, ^21^, ^22^] Along this line, it is thinkable that proto‐RNA existed that was made up from proto‐nucleosides (precursor nucleosides), which rearrange inside the proto‐RNA to RNA. It is also thinkable that these proto‐nucleosides formed a mixed proto‐RNA with a few embedded canonical RNA nucleosides as seed‐nucleotides.^[23]^ The proto‐RNA nucleosides could then rearrange to finally give RNA strands composed exclusively of canonical nucleotides (Figure 1a). Degradation of this rearranged RNA, which would certainly have occurred on the early earth, would have liberated the canonical nucleosides, thus allowing them to be reinserted as seed‐nucleotides into growing proto‐RNA. Here we show that such a concept can indeed give RNA and we provide evidence that the stereo information of the canonical seed‐nucleotides can control the stereochemical outcome of the proto‐RNA to RNA rearrangement. An obstacle for the concept is that in most precursor RNA nucleosides the glycosidic bonds are too labile, so that stable strand formation cannot occur. This is a particular problem for non‐cyclic precursor nucleosides, which typically feature an acidic anomeric H‐atom at the glycosidic N‐atom. Exceptions from this are, however, urea‐based proto‐nucleosides, in which the acidic H‐atom is stabilized by an internal H‐bond.^[24]^

**Figure 1:**
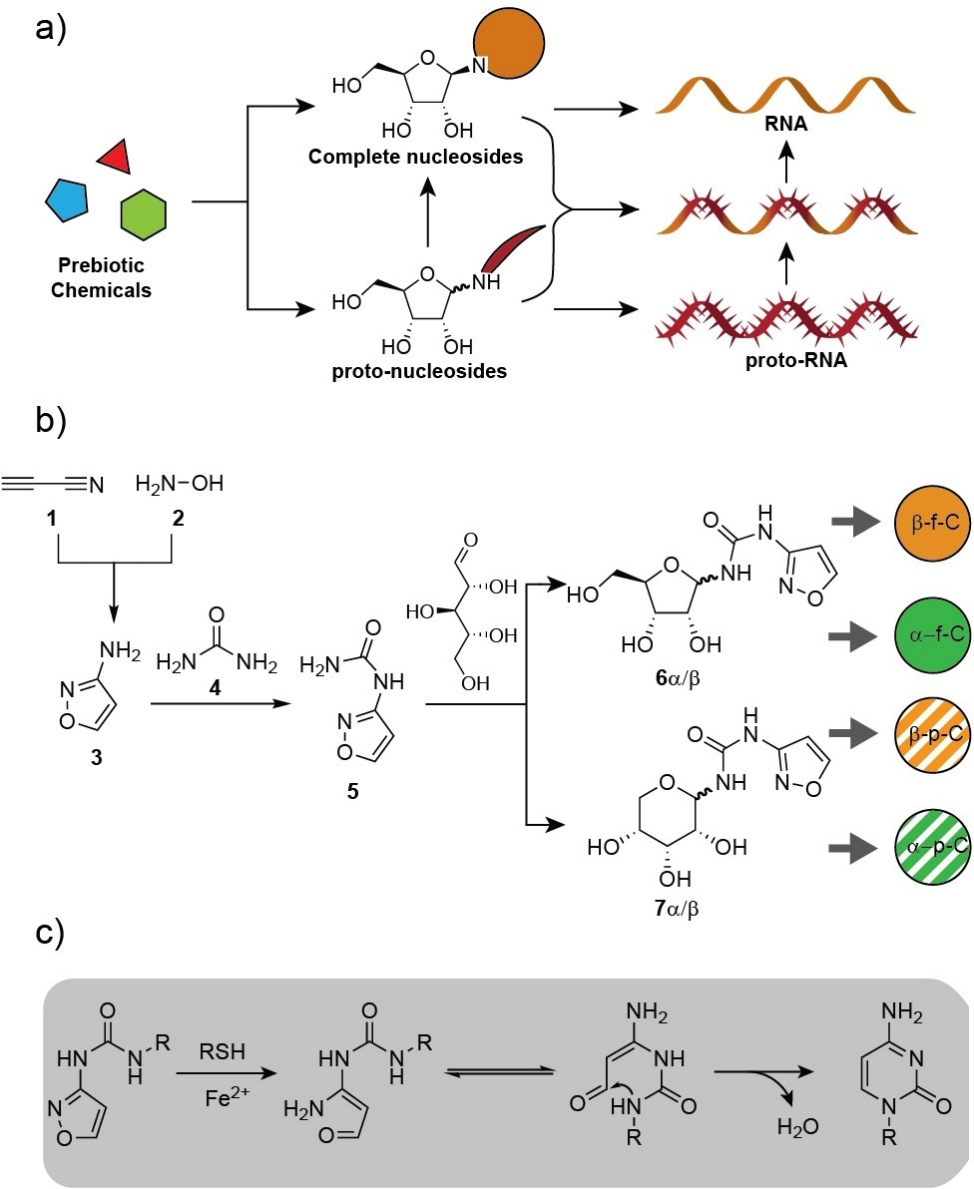
a) Classical prebiotic chemistry provides first the full nucleosides, which are then converted into RNA. Alternatively, proto‐nucleosides could have formed a proto‐RNA or a mixed RNA‐proto‐RNA from which RNA was formed. b) Synthesis of the 3‐urea isoxazole nucleosides **6** and **7**. Upon insertion into RNA, **6**α/β rearranges directly in RNA to give cytidine. c) Mechanism of the reaction.

A recently introduced concept (Figure 1b) of how the pyrimidine nucleoside cytidine could have formed on the early Earth is based on the reaction of cyanoacetylene (**1**) with hydroxylamine (**2**) to give 3‐aminoisoxazole (IO3, **3**).^[25]^ **3** was shown to react with urea (**4**) to give *N*‐isoxazolyl‐urea **5**, which can next react with ribose to furnish the corresponding furanosides (**6**α,β) and pyranosides (**7**α,β). Subsequent reductive N−O bond cleavage with thiols in the presence of catalytic amounts of Fe^2+^ gives cytidines (Figure 1c).[^25^, ^26^, ^27^, ^28^] Here, we report that the initially formed *N*‐isoxazolyl‐urea **5**, if structurally embedded into (RNA)‐proto‐RNA structures, can undergo the N−O cleavage induced rearrangement directly in RNA. This “in‐RNA” reaction creates a new stereocenter. Excitingly, we observe that the configuration of already present canonical seed‐nucleosides controls the stereochemical outcome of the “in‐RNA” cyclization reaction, which leads to an amplification of stereochemical information.

To first investigate the possibility of performing the **6**α/β to C rearrangement directly in‐RNA, we prepared the IO3 phosphoramidites **α‐8** and **β‐8** incorporated both building blocks into the different RNA strands shown in (Table 1). The synthesis of **8** is depicted in Scheme 1. It started with 1‐azidoribose **9**, which was first 3′‐5′‐protected with (tBu)_2_SiCl_2_ (DMF, rt, 78 %) to give **10**. Subsequent protection of the 2′‐OH group with TBS‐Cl (DMF, imidazole, rt, 78 %) provided **11**. This was followed by a one‐pot reduction of the azide to the amine (Pd/C, cat. XPhos, toluene, CO, 5 bar, 60 °C) and reaction of the amine with IO3 **3** to generate the ribofuranoside **12** as the expected α/β‐mixtures. The two IO3 anomers **α‐12** and **β‐12** could be separated by flash column chromatography. The nucleosides were next independently 3′‐5′‐deprotected to **13** (HF‐py, CH_2_Cl_2_ 0 °C) and 5′‐dimethoxytrityl‐protected (DMT‐Cl, pyridine, rt) to give **14**. The 5′‐DMT‐2′‐TBS‐protected IO3 ribosides **14** were finally converted into the phosphoramidites **α‐8** and **β‐8**. While we were initially concerned about the stability of the isoxazole‐ribosides, we learned during the synthesis that all IO3 nucleosides are astonishingly stable. Similar to the previously reported urea nucleosides,^[24]^ we noticed no deglycosylation reaction during the reaction, which is surprising given that the anomeric center features a rather acidic NH‐group.

**Table 1:** Synthesized oligonucleotides with **α**,**β‐8** as needed for this study and their molecular weights determined by MALDI‐TOF mass spectrometry.

Sequence name	Sequence (5′→3′)	Calculated [M−H]^−^	Found (*m/z*)
**S1**	5′‐CUU AC_**β**_ **IO3** CUG A‐3′	3094.4	3094.6
**A‐S1**	5′‐CUU ACA CUG A‐3′	3102.4	3102.3
**G‐S1**	5′‐CUU ACG CUG A‐3′	3118.4	3118.3
**C‐S1**	5′‐CUU ACC CUG A‐3′	3078.7	3078.3
**U‐S1**	5′‐CUU ACU CUG A‐3′	3079.4	3079.5
**A‐R1**	5′‐UCA GAG UAA G‐3′	3205.5	3205.4
**G‐R1**	5′‐UCA GGG UAA G‐3′	3221.5	3221.2
**C‐R1**	5′‐UCA GCG UAA G‐3′	3181.5	3181.8
**U‐R1**	5′‐UCA GUG UAA G‐3′	3182.4	3182.1
**S2**	5′‐_β_C_β_C**IO3**_β_C_β_C**IO3**_β_C_β_C‐3′	2409.4	2409.3
**S3**	5′‐_α_C_α_C**IO3**_α_C_α_C**IO3**_α_C_α_C‐3′	2409.4	2409.1
**S4**	5′‐_α_C_β_C**IO3**_α_C_β_C**IO3**_α_C_β_C‐3′	2409.4	2408.6
**S5**	5′‐**IO3IO3IO3 IO3‐**3′	1222.2	1221.9
**S6**	5′‐**IO3IO3IO3 IO3**_β_C‐3′	1526.2	1526.2
**S7**	5′‐**IO3IO3IO3 IO3**_α_C‐3′	1526.2	1526.2
**S8**	5′‐GGU _**β**_ **IO3**GA CC3′	2538.4	2535.5

**Scheme 1:**
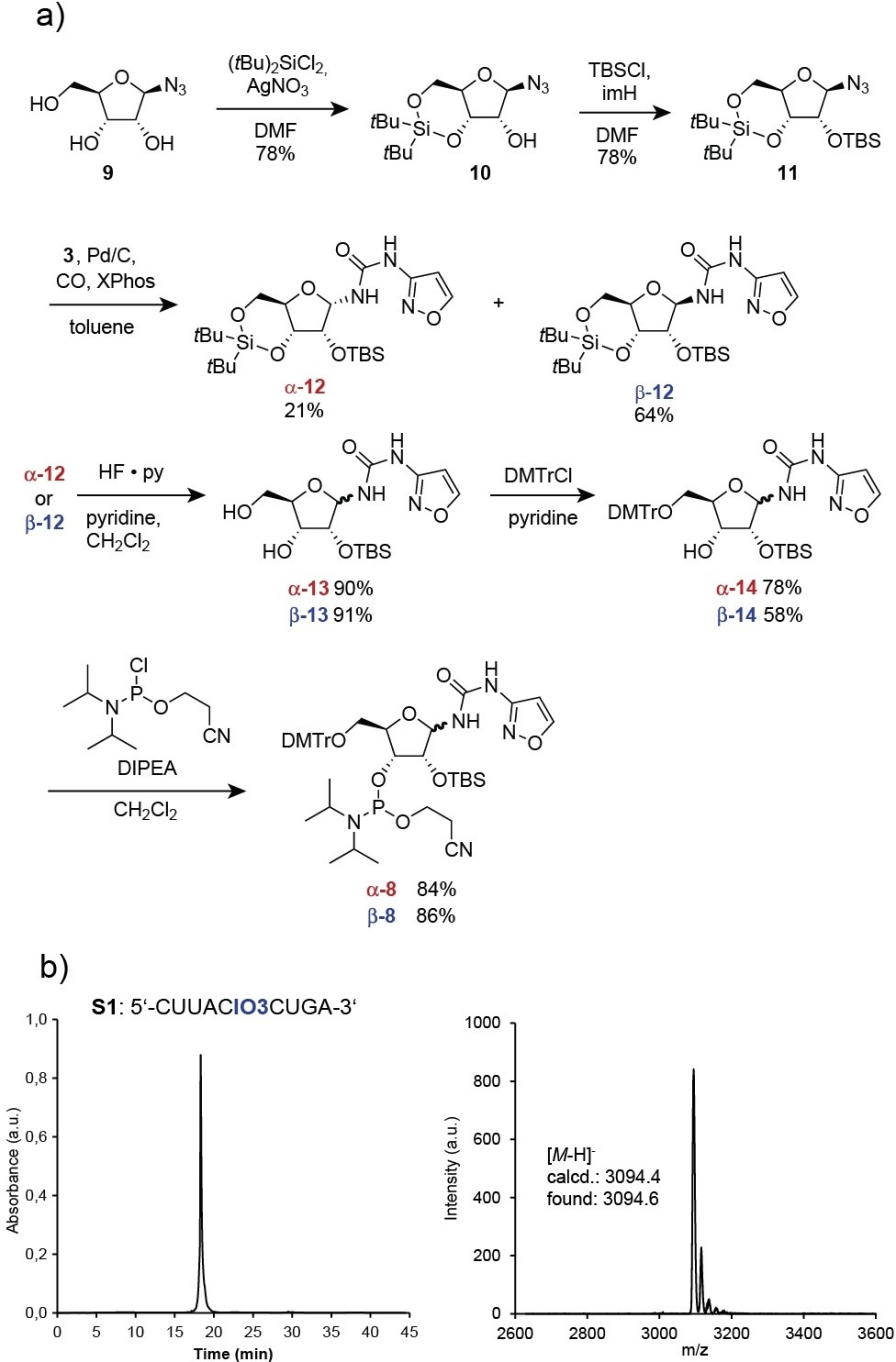
a) Synthesis of the IO3 phosphoramidite α,β‐**8** and conditions. b) HPL‐chromatograms and MALDI‐TOF mass spectra of the oligonucleotide **S1** shown as an example, synthesized with the IO3 phosphoramidite **β‐8**.

To investigate whether an incorporation of the IO3 nucleosides into RNA is possible, we used standard solid phase oligonucleotide chemistry and the phosphoramidites **α‐8** and **β‐8**. The RNA synthesis was indeed possible without large adjustments of the RNA‐synthesis and deprotection protocol. We just had to extend the coupling time of **α‐8** and **β‐8** to 600 s. Deprotection was first achieved with ammonium hydroxide/methylamine (1 : 1) followed by a second deprotection step with TEA⋅3HF. Scheme 1b shows the HPLC‐chromatogram of the synthesized RNA strand **S1** as an example containing a single β‐configured IO3 after purification. The MALDI‐TOF mass spectrum proves the integrity of the obtained RNA **S1**. With this experimental proof for the possible and efficient incorporation of the IO3 nucleoside into RNA, we next used the protocol to prepare the RNA strands **S1**–**S8** containing multiple IO3 nucleosides in mixed sequence contexts.

To investigate the “in‐RNA”‐rearrangement of the IO3 nucleosides to C and to study the stereochemical outcome of the reaction, we first prepared the RNA strand **S2** (Figure 2a) containing two 3‐isoxazole nucleosides embedded into a 8mer containing 6 additional cytidines. For the synthesis, we used the phosphoramidite mixture α/β‐**8**. To investigate the α/β‐ratio after RNA synthesis and HPL‐chromatographic purification of the strand, we digested **S2** after the synthesis down to the individual nucleosides with the New England BioLabs Nucleoside digestion mix (Cat. No. NEB M0649S). The obtained mixture of nucleosides was subsequently analyzed by LC‐HESI‐MS (gray bracket in Figure 2a). Clearly evident is the presence of the β‐C nucleosides, as expected. Besides β‐C, we detected two additional signals for the two isoxazole nucleosides (α‐IO3,β‐IO3). Integration of the signals shows an α/β‐ratio of 1 : 4. Next, we treated the isoxazole‐containing RNA strand **S2** with dithiothreitol (DTT) and catalytic amounts of Fe^2+^ at 90 °C for 2 h. HPL‐chromatographic analysis of the transformation revealed a clean spot‐to‐spot conversion (Figure 2a). While the original RNA strand **S2** had a retention time of 33 min, this signal fully disappeared in favor of a new HPLC‐signal at 23 min, which corresponds to the reduced product strand **S2^red^**. Important is the observation that only one signal is detected and not a set of signals as expected for a diastereoisomeric mixture of products. With the purpose of investigating the stereochemical outcome, we again digested the RNA strand **S2^red^** and analyzed the nucleoside mixture by LC‐HESI‐MS (Figure 2b). This experiment showed to our surprise only one signal for the compound β‐C. Co‐injection of a α‐C confirmed this experimental outcome. α‐C is clearly not formed during the N−O bond cleavage and rearrangement to C in the RNA strand. This result shows that the α/β‐mixture of the IO3 nucleoside in the RNA rearranged exclusively to β‐C by “amplification” of the stereochemical information provided by the seed‐cytidines.

**Figure 2:**
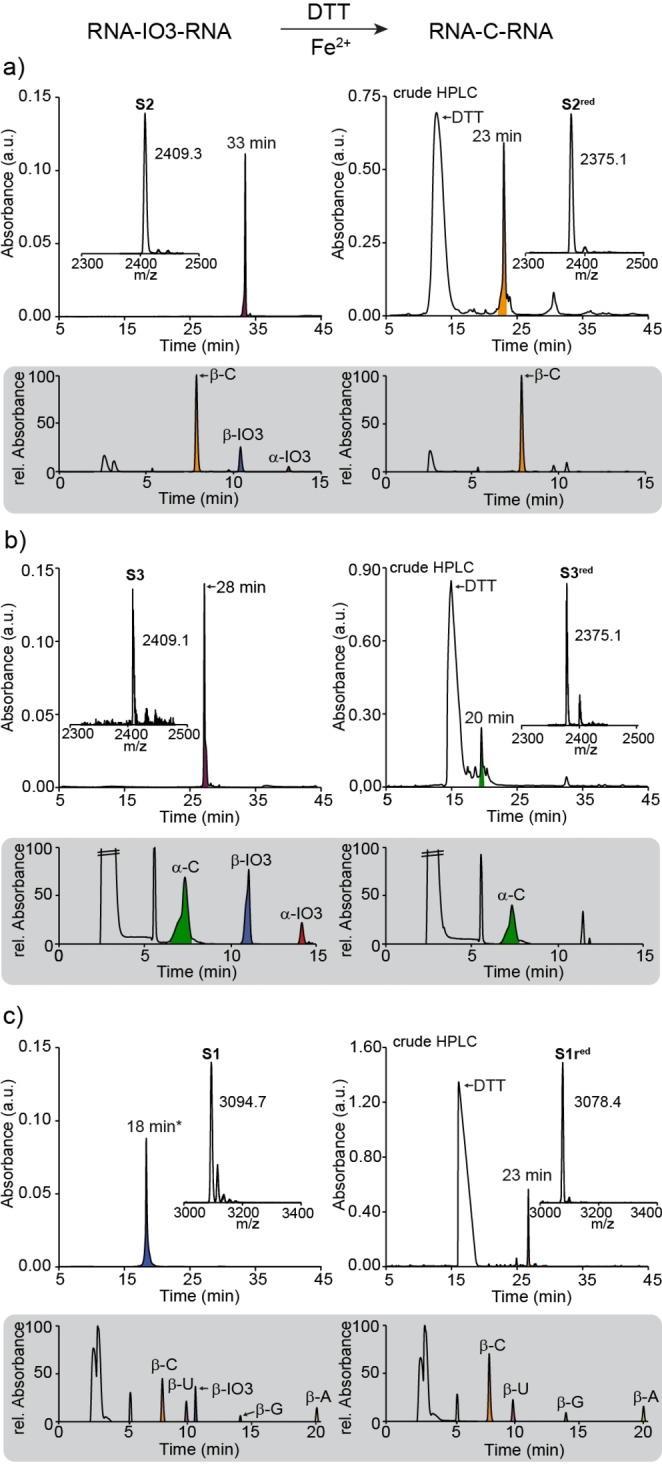
Reaction of the **IO3** containing strands with DTT and Fe^2+^ and HPLC, MALDI, HPLC‐MS (at 223 nm; gray brackets) analyses of the N−O cleavage and cyclization reaction (left: starting material; right: product). a) Result of the β‐homo‐C strand **S2**; b) the α‐homo‐C strand **S3**; c) the strand **S1**. A=adenosine, C=cytidine, G=guanosine, IO3=3‐aminoisoxazole, U=uridine. *HPLC gradient: 0–40 % buffer B.

In order to substantiate this stereochemical amplification step, we next prepared the RNA strand **S3**, in which we replaced the flanking β‐Cs of **S2** by the corresponding α‐Cs. The RNA strand **S3** was again synthesized in excellent quality (Figure 2b). Digestion and LC‐HESI‐MS analysis before the N−O‐bond cleavage shows again the presence of the IO3 nucleosides as an α/β‐mixture. When we opened the N−O bonds of **S3** and analyzed the nucleoside mixture after digestion, we observed to our surprise again only a single HPLC signal. This time only α‐C was detected, showing that now the “in‐RNA”‐rearrangement of the α/β‐mixture of the IO3 furnished only the α‐configured product. Again, the stereo‐information of the anomeric center of the seed‐α‐C nucleosides must have dictated the stereochemical outcome of the cyclization reaction (Figure 2b). We next performed the same reaction with strand **S1** to show that the stereochemical amplification is also achieved with other canonical bases. As expected, we only detected β‐configured C in our product strand **S1^red^** (Figure 2c). These data show that the flanking bases obviously transfer stereochemical information, so that the cyclization reaction proceeds with high stereospecificity. We believe that the information transfer is caused by an optimization of the base stacking forces. A stereochemically homogeneous all‐α or all‐β RNA can establish better base stacking forces in an aqueous solution.

With the purpose of proving that the presence of seed‐nucleosides is a prerequisite for the stereochemical amplification, we performed two control experiments. First, we used the strand **S4**, which is a 8mer oligonucleotide containing both α‐ and β‐seed‐C in a 1 : 1 ratio. Proto‐RNA **S5** in contrast was devoid of any seed‐cytidines. After treatment of these strands with DTT and Fe^2+^, subsequent LC‐HESI‐MS analysis showed as expected the formation of a 1 : 1 mixtures of α/β‐Cs (see Supporting Information).^[29]^

Based on this result, we next designed two 5mers with only one information carrying nucleoside in the form of a single β‐C (**S6**) or α‐C **(S7)** at the 3′ position. LC‐HESI‐MS analysis of the digestion products after N−O bond cleavage and subsequent rearrangement showed us now mixtures of α/β‐C for both strands. However, a clear stereochemical bias is observed. For the β‐C carrying strand **S6^red^** an α/β‐ratio of 1 : 3 is detected and for the α‐C carrying strand **S7^red^** an α/β‐ratio of 2 : 1 was obtained. This shows that one stereochemically defined seed‐nucleotide is already sufficient to control the stereochemical outcome of the cyclization reaction.

For a chemical compound to function as an instructional proto‐nucleoside it is essential that the nucleoside can contribute to the stability of the RNA and that it can encode information.[^30^, ^31^] Therefore, we investigated if the nucleoside β‐IO3 is able to establish informative interactions with one of the other RNA bases. For this study, we synthesized different counter strands for **S1** (**A**/**C**/**G**/**U**‐**R1**) in which we varied the base opposite the IO3 and measured melting points. We also prepared four reference **S1** strand (**A**/**C**/**G**/**U**‐**S1**), in which we replaced the IO3 by one of the other canonical bases (Figure 3a). The duplex with the G : C base pair (**G‐S1**:**C‐R1**) at the respective position features as expected, the highest melting point of 55 °C. The A : U base pair at this position created a duplex (**A‐S1**:**U‐R1**) which melted at *T*_m_=48 °C. For the duplex **G‐S1**:**U‐R1** with a G : U Wobble base pair at the respective position, we measured a melting point of 43 °C. When we investigated the **IO3‐S1**:**R1** base pair, we noted that the IO3:G situation created a duplex **IO3‐S1**:**G‐R1** with a melting point of 45 °C even higher than what was determined for the G : U Wobble base pair. More importantly, the pairing selectivity is remarkable. While the IO3:G containing duplex melts at 45 °C, all other base pairs created duplexes with melting points of 31 °C or even lower, establishing a Δ*T*_m_ of minimum 14 °C. This is an unusual discrimination between the productive IO3:G situation and all other base pairing possibilities.

**Figure 3:**
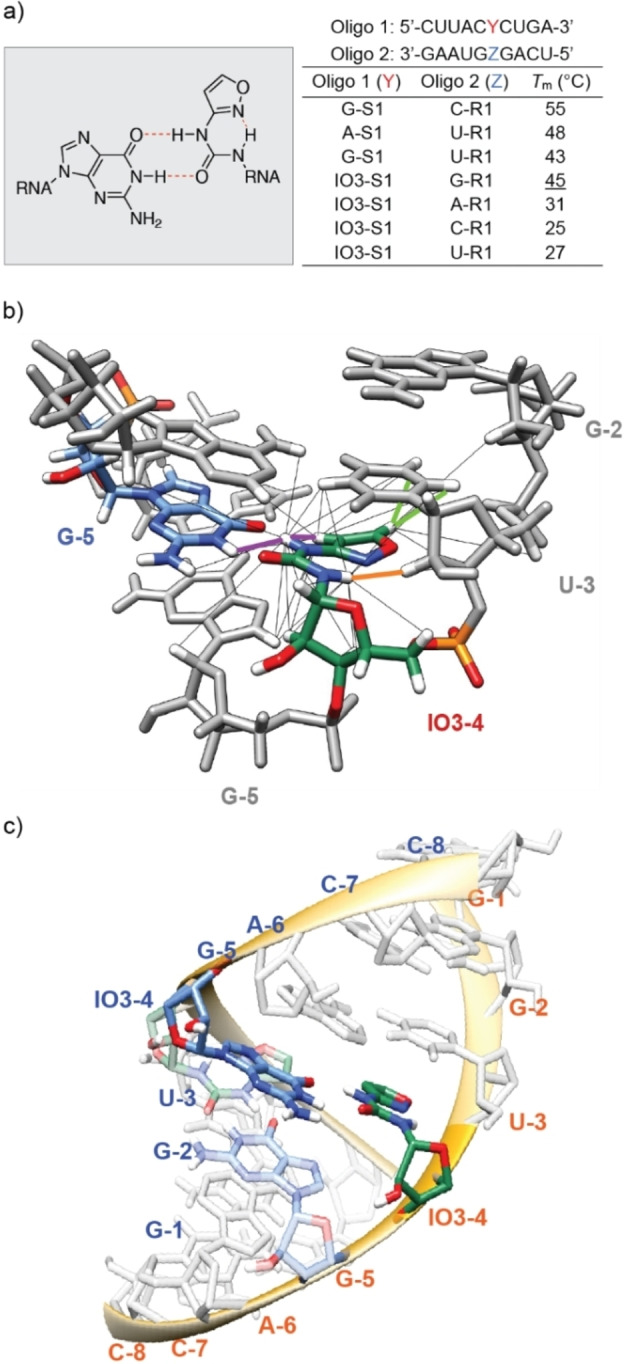
a) Chemical structure and base pairing properties of a IO3:G base pair and summary of *T*_m_ analyses for canonical oligonucleotides and oligonucleotides containing IO3. Solutions were buffered with 10 mM sodium phosphate (pH 7) and 150 mM NaCl. b) Predicted orientation of the IO3 with all the NOE contacts (black lines) and the most important contacts for the structure determination highlighted (IO3‐4H3:G5H1 (purple), IO3‐4H1:U3H2′ (orange), IO3‐4H7:U3H5 and IO3‐4H7:U3H6 (green)). c) Structural model of **S8**, showing the non‐canonical base pairing between G‐5 (blue) and IO3–4 (green).

In order to investigate the reason for the constructive interaction of the IO3:G base pair, we designed two palindromic 8mer RNA strands **S8** and **Can_8_** (see Supporting Information), which upon annealing result in formation of either G : U or IO3:G wobble base pairs at the central two positions in each duplex, respectively (GGU**X**GACC). These strands were prepared in mg‐quantities and the formed duplexes were analyzed by NMR spectroscopy (Figure S7). The high chemical shift similarity observed across the two samples, both in homo‐ and heteronuclear experiments, showed us that both palindromes form double‐strands with an overall A‐like conformation which is typical for RNA duplexes (Figure S7–S11, Table S1). 2D ^1^H–^1^H NOESY was next employed to elucidate the conformation of the IO3 base (Figure 3b, see Supporting Information for extended methods). The most probable orientation was determined by analysis of the cross peaks in the imino region. A considerable upfield shift of the G‐5H1 proton can be observed (from 12.1 to 11.0 ppm), indicating the presence of a weak hydrogen bond of G‐5 to IO3–4 (Figure S11). The intense cross peak between G‐5H1 and IO3‐4H3 (purple line in Figure 3b), which could be detected even at a short mixing time of 40 ms, shows that the two bases face each other. This observation implies that IO3‐4H3 points in the direction of the second strand and hence away from the sugar phosphate backbone. A cross peak between protons G‐5H1 and IO3‐4H8 confirms that the isoxazole‐ring of IO3–4 point towards G‐5. A cross peak with the sugar proton U‐3H2′ (orange line in Figure 3b) furthermore suggests that the IO3‐4H1 protrudes towards the sugar phosphate backbone, similarly to H8 protons in adenine and guanine or the H5 protons of cytosine or uracil. Finally, the strong cross peaks between IO3‐4H7 and the aromatic protons of U‐3 (green lines in Figure 3b) and the weaker cross peaks of IO3‐4H8 to these protons indicate that IO3‐4 and U‐3 are stacked, arguing that the aromatic ring is oriented in the direction of the major groove.

This leads to an orientation such that IO3‐4H1 can form an intramolecular hydrogen bond with IO3‐4O4. Such an arrangement has the carbonyl group pointing towards the guanine base, allowing, in line with the melting point studies, a productive base‐pairing interaction with G‐5H1. Overall, this analysis establishes that IO3 adopts a flat conformation in which the NH‐CO‐unit of the base pairs points towards the G counter base to form constructive H‐bonds. It is the urea substructure, which establishes H‐bonds with the G counter base. The fact that only two H‐bonds can form, is consistent with the lower melting point. The 2D ^1^H‐^1^H NOESY data were further used to perform structure calculations (Figure 3c). The low‐energy structural model obtained for the modified 8mer shows that the IO3:G base‐pair is sterically perfectly embedded within the double‐stranded structure. Interesting is the fact that the internal H‐bond between the isoxazole N and the urea N−H is strongly stabilizing the structure. The IO3‐nucleoside forms 2 hydrogen bond with the G counter base, which upon rearrangement to C increases to 3, which goes in hand with a further stabilization of the base pair. This could have been the evolutionary driving force.

In summary, we show that isoxazole‐urea nucleosides that are formed under prebiotically plausible conditions from just cyanoacetylene, hydroxylamine, urea and ribose^[25]^ can form a base pair with G that has a stability comparable to a U : G Wobble base pair, based on two instructive H‐bonds (Figure 3a). More importantly, the IO3:G base pair forms rather selectively because all other combinations (IO3:A/C/U) are substantially weaker. We show that the N−O bond of the isoxazole nucleoside can be rapidly cleaved within a proto‐RNA strand with thiols and catalytic amounts of Fe^2+^. This cleavage induces a cyclization‐elimination cascade (Figure 1c) that gives β‐ or α‐cytidine. Importantly, the presence of α‐ or β‐seed‐nucleosides defines the stereochemical outcome of the rearrangement reaction.

We do not know whether such IO3‐nucleosides were once components of an early proto‐RNA world, but it is generally assumed that RNA was preceded by simpler precursor structures.[^16^, ^30^] Here, we show that isoxazole nucleosides are excellent candidates for such precursor nucleosides and proto‐RNAs. Because the proto‐nucleosides can rearrange to C also just in solution, it is reasonable to assume that IO‐nucleosides and the canonical α‐/β‐nucleosides might have co‐existed to form proto‐RNA strands containing a few canonical seed cytidines. These nucleosides may then have controlled the stereochemical outcome of the rearrangement that converted the G:IO3 base pair into the better pairing G : C base pair. Because α‐ and β‐Cs form with the same probability, our data provide no solution of the question of how homo‐chirality was established.^[32]^ The data shows, however, that stereochemical information could have been amplified by “in‐RNA reactions” of precursor nucleosides to canonical nucleosides and this can stimulate thought about the origin of homo‐chirality.

## Conflict of interest

The authors declare no conflict of interest.

## Supporting information

As a service to our authors and readers, this journal provides supporting information supplied by the authors. Such materials are peer reviewed and may be re‐organized for online delivery, but are not copy‐edited or typeset. Technical support issues arising from supporting information (other than missing files) should be addressed to the authors.

Supporting Information

## Data Availability

The data that support the findings of this study are available in the supplementary material of this article.
