# Supplementary material for: Isoxazole Nucleosides as Building Blocks for a Plausible Proto‐RNA
Source: Angew Chem Int Ed Engl. 2022 Oct 11;61(45):e202211945. doi: 10.1002/anie.202211945 (PMC9828505; doi:10.1002/anie.202211945)
Supplement: Supplementary file 1 — Supporting Information [file ANIE-61-0-s001.pdf]

Angewandte Chemie
Eine Zeitschrift der Gesellschaft Deutscher Chemiker

Supporting Information

Isoxazole Nucleosides as Building Blocks for a Plausible Proto-RNA

*F. Xu, A. Crisp, T. Schinkel, R. C. A. Dubini, S. Hübner, S. Becker, F. Schelter, P. Rovó, T. Carell**

Table of Contents

Table of Contents 2
General Experimental Methods 3
Synthesis and characterization of the Phosphoramidite Building Blocks 3
Synthesis and purification of oligonucleotides 7
UV Melting Curve Measurements 12
In-strand cytidine formation reactions 12
Digestion and LC-HESI-MS analysis 13
High-resolution NMR studies of IO-containing RNA oligonucleotides 15
Molecular modelling of IO-containing RNA oligonucleotides 16
Schematic sample representation 17
Homo- and heteronuclear correlation spectra 17
Chemical shift assignment tables 20
NMR spectra of synthesized compounds 21
References 29

General Experimental Methods

Chemicals were purchased from Sigma-Aldrich, TCI, Fluka, ABCR, Carbosynth or Acros organics and used without further purification. The solvents were of reagent grade or purified by distillation. Reactions and chromatography fractions were monitored by qualitative thin-layer chromatography (TLC) on silica gel F_{254} TLC plates from Merck KGaA. Flash column chromatography was performed on Silicagel 60 (40-63 µm) silica gel from Macherey-Nagel. Reactions were conducted under a positive pressure of dry nitrogen in oven-dried glassware, and at ambient room temperature, unless otherwise specified. NMR spectra were recorded on Bruker AVIIIHD 400 (400 MHz) or Bruker Avance III (800 MHz) spectrometers. ^{1}H NMR shifts were calibrated to the residual solvent resonances: DMSO-*d*$_6$ (2.50 ppm), CD_3OD (4.87 ppm), Acetone-*d*$_6$ (2.05 ppm), $CDCl_3$ (7.26 ppm). ^{13}C NMR shifts were calibrated to the residual solvent: DMSO-*d*$_6$ (39.52 ppm), CD_3OD (49.00 ppm), $CDCl_3$ (77.16 ppm), Acetone-*d*$_6$ (29.84 ppm). All NMR spectra were analyzed using the program MestRE NOVA 10.0.1 from Mestrelab Research S. L. Normal resolved mass spectra were measured on a LTQ FT-ICR by Thermo Finnigan GmbH. High resolution mass spectra were measured by the analytical section of the Department of Chemistry of the Ludwig-Maximilians-Universität München on the following spectrometers (ionization mode in brackets): MAT 95 (EI) and MAT 90 (ESI) from Thermo Finnigan GmbH, unless otherwise specified. IR spectra were recorded on a PerkinElmer Spectrum BX II FT-IR system.

Synthesis and characterization of the Phosphoramidite Building Blocks

2-*O*-*tert*-butyldimethylsilyl-3,5-*O*-(di-*tert*-butylsilandiyl)-1-β-D-ribofuranosy-azide (11)

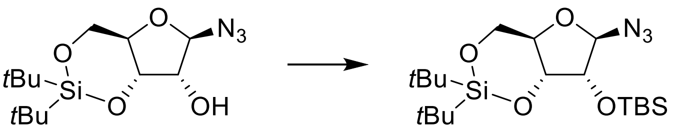

10 **11**

10[1] (10.9 g, 34.5 mmol, 1.00 eq.) was dissolved in DMF (36 mL) imidazole (11.8 g, 173 mmol, 5.00 eq.) and TBS-Cl (6.25 g, 41.5 mmol, 1.20 eq.) were added and the mixture was stirred at rt for 16 h. The solvent was evaporated, the residue was taken up in EtOAc (50 mL) and sat. $NaHCO_3$-solution (50 mL) and extracted with EtOAc (2×50 mL). The combined organic layers were dried over Na_2SO_4 and concentrated in vacuo. The crude product was purified by flash column chromatography (silica, iHex:EtOAc = 100:1) to yield **11** as colorless crystals (11.6 g, 26.9 mmol, 78%).

***R*$_f$** = 0.43 (iHex:EtOAc = 100:1);**^{1}H NMR** (400 MHz, $CDCl_3$): δ (ppm) = 5.07 (s, 1H, 1-H), 4.42 (dd, *J* = 9.1, 5.1 Hz, 1H, 5-H), 4.12 (ddd, *J* = 10.9, 9.6, 5.1 Hz, 1H, 4-H), 4.01 (d, *J* = 4.1 Hz, 1H, 2-H), 3.95 – 3.88 (m, 2H, 3-H, 5-H), 1.05 (s, 9H, Si-C-CH_3), 1.00 (s, 9H, Si-C-CH_3), 0.91 (s, 9H, Si-C-CH_3), 0.13 (s, 3H, Si-CH_3), 0.12 (s, 3H, Si-CH_3);**^{13}C NMR** (101 MHz, $CDCl_3$): δ (ppm) = 96.19 (C1), 76.33 (C4), 75.98 (C2), 74.65 (C3), 68.42 (C5), 27.58 (Si-C-CH_3), 27.15 (Si-C-CH_3), 25.98 (Si-C-CH_3), 22.83 (Si-C), 20.47 (Si-C), 18.50 (Si-C), -4.25 (Si-CH_3), -5.06 (Si-CH_3); **IR** (cm^{-1}): ṽ = 4029 (w), 3843 (w), 3775 (w), 2933 (s), 2884 (m), 2859 (s), 2386 (w), 2205 (w), 2109 (s), 1472 (m), 1387 (m), 1362 (m), 1297 (w), 1253 (s), 1167 (m), 1142 (s), 1049 (s), 998 (s), 925 (m), 880 (m), 826 (s), 776 (s), 753 (w); **HRMS** (EI): calcd. for $C_{18}H_{36}N_3O_4Si_2^+$ $[M]^+$: 414.2239; found: 414.2252.

N-isoxazol-3-yl-*N*'-(1'-(2'-*O*-*tert*-butyldimethylsilyl-3',5'-*O*-(di-*tert*-butylsilandiyl)-β-D-ribofuranosyl))-urea (12)

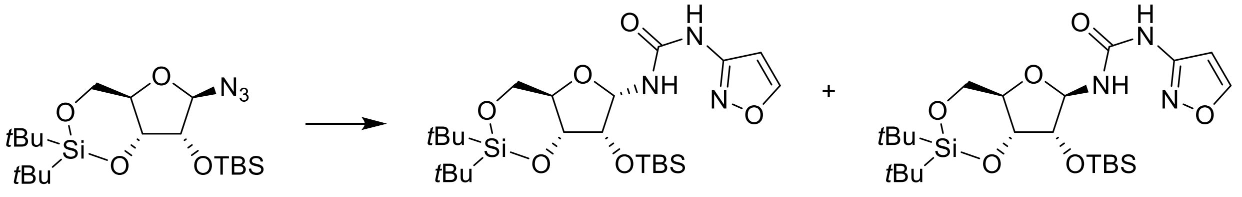

11 **α-12** **β-12**

11 (1.01 g, 2.36 mmol, 1.00 eq.) and 3-aminoisoxzole (209 µL, 2.83 mmol, 1.20 eq.) were dissolved in dry toluene (23 mL). Palladium on carbon (10%, 126 mg, 118 µmol, 0.05 eq.) and XPhos (113 mg, 236 µmol, 0.1 eq.) were added to the solution. The reaction mixture was stirred at 60°C under CO-atmosphere (5 bar) for 18 h. The catalyst was filtered off with a pad of $MgSO_4$ and washed with toluene. The filtrate was concentrated under reduced pressure and the crude product was purified by flash column chromatography (silica, *i*Hex:EtOAc = 4:1) to yield **α-12** (255 mg, 496 µmol, 21%) and **β-12** (775 mg, 1.51 mmol, 64%) as colorless solids.

α-12:
***R*f** = 0.19 (*i*Hex:EtOAc = 4:1); **^{1}H NMR** (600 MHz, $CDCl_3$): δ (ppm) = 8.42 (s, 1H, NH), 8.20 (d, *J* = 1.8 Hz, 1H, 5-H), 6.43 (s, 1H, 4-H), 5.90 (dd, *J* = 9.2, 4.1 Hz, 1H, 1'-H), 4.39 – 4.34 (m, 2H, 2'-H, 5'-H), 4.04 (td, , *J* = 10.0, 5.2 Hz, 1H, 4'-H), 3.94 (dd, *J* = 9.7, 4.5 Hz, 1H, 3'-H), 3.86 – 3.82 (m, 1H, 5'-H), 1.06 (s, 9H, Si-C-CH_3), 1.02 (s, 9H, Si-C-CH_3), 0.96 (s, 9H, Si-C-CH_3), 0.18 (s, 3H, Si-CH_3), 0.17 (s, 3H, Si-CH_3); **^{13}C NMR** (151 MHz, $CDCl_3$): δ (ppm) = 158.3 (C5), 157.7 (C3), 153.8 (C=O), 98.2 (C4), 82.2 (C1'), 77.7 (C3'), 72.5 (C4'), 71.0 (C4'), 68.3 (C5'), 27.5 (C), 27.1 (Si-C-CH_3), 26.2 (Si-C-CH_3), 22.7 (Si-C-CH_3), 22.7 (Si-C), 20.3 (Si-C), 18.6 (Si-C), -4.3 (Si-CH_3), -4.9 (Si-CH_3); **IR** (cm^{-1}): $\tilde{\nu}$ = 3342 (w), 2931 (m), 2859 (m), 1707 (s), 1590 (s), 1504 (s), 1468 (m), 1389 (w), 1260 (w), 1209 (w), 1174 (w), 1114 (m), 1053 (s), 964 (m), 905 (w). 827 (s), 773 (m), 705 (w); **HRMS** (ESI): calcd. for $C_{23}H_{44}N_3O_6Si_2^+$ [M+H]$^+$: 514.27632, found: 514.27643.

β-12:
***R*f** = 0.21 (*i*Hex:EtOAc = 4:1); **^{1}H NMR** (600 MHz, $CDCl_3$): δ (ppm) = 8.85 (s, 1H, NH), 8.20 (d, ^3J = 1.8 Hz, 1H, 5-H), 6.27 (s, 1H, 4-H), 5.47 (d, ^3J = 8.5 Hz, 1H, 1'-H), 4.40 (dd, ^2J = 9.2, ^3J = 5.0 Hz, 1H, 5'-H), 4.21 (d, ^3J = 4.7 Hz, 1H, 2'-H), 4.04 (ddd, ^3J = 10.4 Hz, ^3J = 9.5 Hz, ^3J = 5.1 Hz, 1H, 4'-H), 3.93 - 3.87 (m, 2H, 3'-H, 5'-H), 1.07 (s, 9H, Si-C-CH_3), 1.02 (s, 9H, Si-C-CH_3), 0.93 (s, 9H, Si-C-CH_3), 0.15 (s, 3H, Si-CH_3), 0.13 (s, 3H, Si-CH_3); **^{13}C NMR** (151 MHz, $CDCl_3$): δ (ppm) = 158.4 (C5), 158.1 (C=O), 153.7 (C3), 98.2 (C4), 89.4 (C1'), 77.1 (C3'), 76.2 (C2'), 73.7 (C4'), 68.6 (C5'), 27.6 (Si-C-CH_3), 27.2 (Si-C-CH_3), 26.1 (Si-C-CH_3), 22.9 (Si-C), 20.50 (Si-C), 18.52 (Si-C), -4.2 (Si-CH_3), -4.9 (Si-CH_3); **IR** (cm^{-1}): $\tilde{\nu}$ = 3340 (w), 2931 (m), 2858 (m), 1706 (s), 1593 (m), 1503 (m), 1468 (m), 1388 (w), 1260 (w), 1113 (m), 1054 (s), 963 (s), 827 (s), 774 (s), 705 (m); **HRMS** (ESI): calcd. for $C_{23}H_{44}N_3O_6Si_2^+$ [M+H]$^+$: 514.27632, found: 514.27662.

N-isoxazol-3-yl-*N'*-(1'-(2'-*O*-*tert*-butyldimethylsilyl)-β-D-ribofuranosyl)-urea (13)

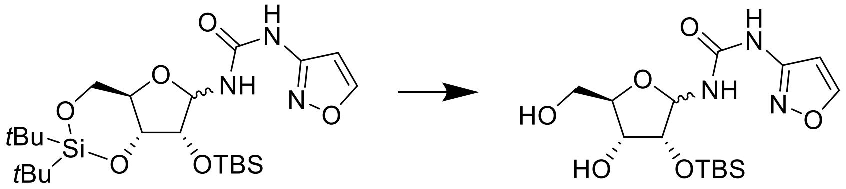

α-12
β-12

α-13
β-13

α-12 or **β-12** (1.00 eq.) was dissolved in DCM and treated with Pyridine (0.01 M) and HF (70% in Pyridine, 5.00 eq.) at 0°C. The resulting mixture was stirred for 4 h and afterwards quenched by the addition of methoxytrimethylsilane. The solution was diluted with DCM and washed with sat. $NaHCO_3$. The aqueous phase was extracted with DCM (3×). The combined organic layers were washed with Brine, dried over Na_2SO_4, and concentrated under reduced pressure. The crude product was purified by flash column chromatography (silica, DCM:MeOH = 100:5) to yield **13** as a colorless foam.

β-13
yield: 91%; ***R*f** = 0.43 **(**DCM:MeOH = 100:5); **^{1}H NMR** (600 MHz, $CDCl_3$): δ (ppm) = 9.29 (s, 1H, NH), 8.21 (d, *J* = 1.8 Hz, 1H, 5-H), 6.50 (s, 1H, 4-H), 5.24 (t, *J* = 6.6 Hz, 1H, 1'-H), 4.54 (t, *J* = 5.3 Hz, 1H, 2'-H), 4.20 (dt, *J* = 5.4, 3.4 Hz, 1H, 3'-H), 4.08 (t, ^3J = 2.6 Hz, 1H, 4'-H), 3.86 (dd, *J* = 9.6, 2.3 Hz, 1H, 5'-H), 3.70 (m , 2H, 5'-H, 5'-OH), 2.81 (d, *J* = 3.7 Hz, 1H, 3'-OH), 0.91 (s, 9H, Si-C-CH_3), 0.13 (s, 3H, Si-CH_3), 0.12 (s, 3H, Si-CH_3); **^{13}C NMR** (151 MHz, $CDCl_3$): δ (ppm) = 158.6 (C5), 158.2 (C=O), 154.7 (C3), 98.3 (C4), 87.4 (C1'), 84.8 (C4'), 74.6 (C2'), 71.9 (C3'), 62.9 (C5'), 25.9 (Si-C-CH_3), 18.18 (Si-C), -4.49 (Si-CH_3), -4.91 (Si-CH_3); **IR** (cm^{-1}): $\tilde{\nu}$ = 3304 (m), 2928 (m), 2856 (m), 1676 (s), 1594 (s), 1531 (s), 1471 (m), 1373 (w), 1254 (w), 1126 (w), 996 (m), 903 (w), 837 (s), 780 (s), 667 (w); **HRMS** (ESI): calcd. for $C_{15}H_{27}N_3O_6Si^+$ [M+H]$^+$: 374.17419, found: 374.17397.

α-13:
yield: 90%; *R*f = 0.42 (DCM:MeOH = 100:5); **^{1}H NMR** (600 MHz, $CDCl_3$): δ (ppm) = 8.46 (s, 1H, NH), 8.20 (d, *J* = 1.8 Hz, 1H, 5-H), 6.42 (brs, 1H, 4-H), 5.80 (dd, *J* = 8.4 Hz, 5.6 Hz, 1H, 1'-H), 4.33 (t, *J* = 5.6 Hz, 1H, 2'-H), 4.13 – 4.11 (m, 1H, 3'-H), 4.09 (q, *J* = 3.3 Hz, 1H, 4'-H), 3.82 (d, *J* = 12.3 Hz, 1H, 5'-H), 3.70 – 3.64 (m, 1H, 5'-H), 2.77 (d, *J* = 9.2 Hz, 1H, 3'-OH), 2.40 (d, *J* = 7.9 Hz, 1H, 5'-OH), 0.93 (s, 9H, Si-C-CH_3), 0.16 (s, 3H, Si-CH_3), 0.12 (s, 3H, Si-CH_3); **^{13}C NMR** (151 MHz, $CDCl_3$): δ (ppm) = 158.5 (C5), 158.0 (C3), 154.4 (C=O), 98.2 (C4), 83.5 (C4'), 81.9 (C1'), 72.0 (C3'), 71.6 (C2'), 62.6 (C5'), 25.9 (Si-C-CH_3), 18.3 (Si-C), -4.60 (Si-CH_3), -4.91 (Si-CH_3); **IR** (cm^{-1}): $\tilde{\nu}$ = 3295 (m), 2926 (m), 1678 (s), 1594 (s), 1532 (s), 1472 (m), 1368 (w), 1248 (w), 1117 (w), 995 (m), 839 (s), 782 (s); **HRMS** (ESI): calcd. for $C_{15}H_{27}N_3O_6Si^+$ [M+H]$^+$: 374.17419, found: 374.17463.

***N*-isoxazol-3-yl-*N'*-(1'-(5'-*O*-(4,4'-dimethoxytrityl)-2'-*O*-*tert*-butyldimethylsilyl)-β-D-ribofuranosyl)-urea (14)**

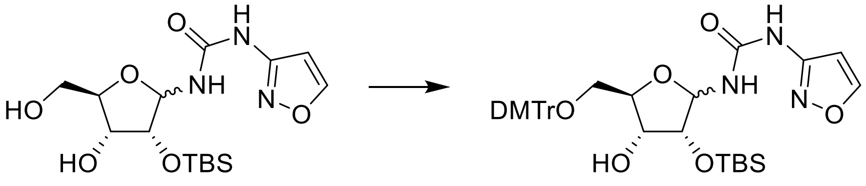

α-13
β-13

α-14
β-14

α-13 or **β-13** (1.00 eq.) was dissolved in pyridine and pre-dried DMT-Cl (1.40 eq.) was added. The resulting mixture was stirred at room temperature for 18 h. The Reaction was quenched by the addition of a few drops of methanol. Afterwards the solvent was evaporated *in vacuo* and the crude product was purified by column chromatography (silica, *i*Hex:EE = 2:1 + 0.1% pyridine) to yield **14** as a yellow foam.

β-14
yield: 58%; ***R*f** = 0.31 (*i*Hex:EtOAc = 2:1);**^{1}H NMR** (800 MHz, Acetone-*d*$_6$): δ (ppm) = 8.87 (s, 1H, NH), 8.54 (d, *J* = 1.5 Hz, 1H, 5-H), 7.51 – 7.49 (m, 2H, Ar-H), 7.38 – 7.35 (m, 4H, Ar-H), 7.31 – 7.28 (m, 2H, Ar-H), 7.25 – 7.21 (m, 2H, Ar-H), 7.18 – 7.16 (m, 2H, Ar-H, NH), 7.14-7.12 (m, 1H, Ar-H), 6.75 (d, *J* = 1.5 Hz, 1H, 4-H), 5.57 (dd, *J* = 9.5 Hz, *J* = 5.5 Hz, 1H, 1'-H), 4.28 (t, *J* = 5.3 Hz, 1H, 2'-H), 4.06 (td, *J* = 4.9 Hz, 3.7 Hz, 1H, 3'-H), 3.99 (q, *J* = 3.8 Hz, 1H, 4'-H), 3.77 (s, 6H, O-CH$_3$), 3.56 (d, *J* = 4.9 Hz, 1H, 3'-OH), 3.31 (dd, *J* = 10.2 Hz, 3.2 Hz, 1H, 5'-H), 3.10 (dd, *J* = 10.2; 4.2 Hz, 1H, 5'-H), 0.93 (s, 9H, Si-C-CH$_3$), 0.18 (s, 3H, Si-CH$_3$) 0.17 (s, 3H, Si-CH$_3$); **^{13}C NMR** (201 MHz, Acetone-*d*$_6$): δ (ppm) = 159.93 (C3) 159.57 (C5), 159.49 (ArC-OCH$_3$), 154.04 (C=O), 146.21 (ArC), 136.97 (ArC), 136.78 (ArC), 131.02 (ArC), 139.76 (ArC), 129.07 (ArC), 128.59 (ArC), 127.50 (ArC), 126.12 (ArC), 113.87 (ArC), 99.08 (C4), 86.84 (C-Ar$_3$), 86.03 (C1'), 83.13 (C4'), 77.31 (C2'), 72.65 (C3'), 65.34 (C5'), 55.47 (O-CH$_3$), 26.21 (Si-C-CH$_3$), 18.89 (Si-C), -4.45 (Si-CH$_3$), -4.52 (Si-CH$_3$); **IR** (cm^{-1}): $\tilde{\nu}$ = 3306 (w), 2952 (m) 2929 (m), 2361 (w), 1708 (m), 1606 (m), 1507 (s), 1483 (m), 1363 (m), 1300 (m), 1249 (s), 1175 (s), 1030 (s), 992 (m), 909 (m), 833 (s), 780 (s), 701 (m); **HRMS** (ESI): calcd. for $C_{36}H_{45}N_3O_8Si^+$ [M+H]$^+$: 674.29032, found: 674.29055.

α-14:
yield: 78%; ***R*f** = 0.30 (*i*Hex:EtOAc = 2:1)**; ^{1}H NMR** (800 MHz, Acetone-*d*$_6$): δ (ppm) = 9.10 (s, 1H, NH), 8.51 (d, *J* = 1.7 Hz, 1H, 5-H), 7.53 – 7.45 (m, 2H, Ar-H), 7.37 – 7.33 (m, 4H, Ar-H), 7.33 – 7.28 (m, 2H, Ar-H), 7.25 – 7.20 (m, 1H, Ar-H), 6.91 – 6.86 (m, 4H, Ar-H), 6.79 (s, 1H, 4-H), 5.89 (dd, *J* = 9.5, 5.6 Hz, 1H, 1'-H), 4.57 – 4.52 (m, 1H, 2'-H), 4.11 (dt, *J* = 4.9, 3.2 Hz, 1H, 3'-H), 4.08 (q, *J* = 3.6 Hz, 1H, 4'-H), 3.88 (d, ^{3}J = 3.5 Hz, 1H, 3'-OH), 3.79 (s, 6H, O-CH$_3$), 3.28 (dd, *J* = 10.2, 3.6 Hz, 1H, 5'-H), 3.06 (dd, *J* = 10.2, 3.7 Hz, 1H, 5'-H), 0.93 (s, 9H, Si-C-CH$_3$), 0.17 (s, 3H, Si-CH$_3$), 0.15 (s, 3H, Si-CH$_3$; **^{13}C NMR** (201 MHz, Acetone-*d*$_6$): δ (ppm) = 159.6 (C3), 159.6 (C5), 159.6 (ArC-OCH$_3$), 154.1 (C=O), 137.0 (ArC), 136.8 (ArC), 131.0 (ArC), 129.0 (ArC), 128.6 (ArC), 127.5 (ArC), 113.9 (ArC), 99.2 (C4), 86.9 (C-Ar$_3$), 83.1 (C4'), 82.3 (C1'), 73.6 (C3'), 72.8 (C2'), 65.0 (C5'), 55.5 (O-CH$_3$), 55.5 (O-CH$_3$), 26.3 (Si-C-CH$_3$), 18.8 (Si-C), -4.6 (Si-CH$_3$), -4.7 (Si-CH$_3$); **IR** (cm^{-1}): $\tilde{\nu}$ = 3314 (w), 2953 (m) 2930 (m), 2361 (w), 1708 (m), 1606 (m), 1506 (s), 1484 (m), 1363 (m), 1300 (m), 1250 (s), 1176 (s), 1032 (s), 994 (m), 910 (m), 835 (s), 780 (s), 702 (m); **HRMS** (ESI): calcd. for $C_{36}H_{45}N_3NaO_8Si^+$ [M+Na]$^+$: 698.28736, found: 698.28764.

***N*-isoxazol-3-yl-*N'*-(1'-(5'-*O*-(4,4'-dimethoxytrityl)-3'-*O*-[2-cyanoethoxy(diisopropylamino)phosphino]-2'-*O*-*tert*-butyldimethylsilyl)-β-D-ribofuranosyl)-urea (8)**

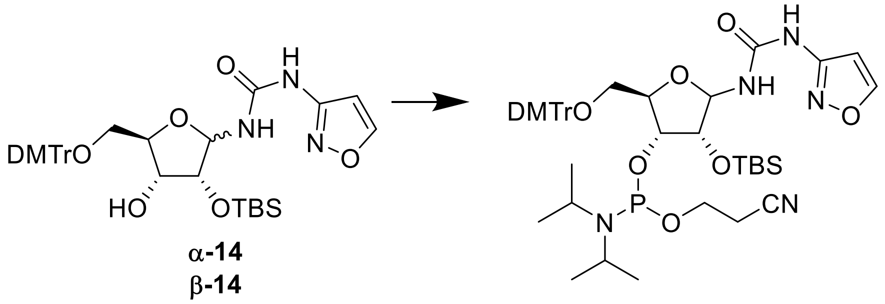

α-14
β-14

α-8
β-8

α-14 or **β-14** (1.00 eq.) was pre-dried by lyophilization from benzene (3×) and dissolved in DCM at 0 °C under argon atmosphere. DIPEA (4.00 eq.) and 2-cyanoethyl *N,N*-diisopropylchlorophosphoramidite (2.50 eq.) were added dropwise. The resulting solution was stirred at room temperature for 3 h and subsequently quenched by the addition of sat. $NaHCO_3$ solution . The aqueous layer was extracted with DCM (3x). The combined organic layers were washed with brine, dried over Na_2SO_4, and concentrated *in vacuo*. The residue was purified by flash column chromatography (silica, *i*Hex:EE = 2:1 + 0.1% pyridine, HPLC grade solvents) to yield **8** as mixture of diastereoisomers, as a colorless solid.

β-8:
yield: 86%; ***R*f** = 0.40, 0.42 (*i*Hex:EtOAc = 2:1); **^{31}P NMR** (162 MHz, Acetone-d_6): δ (ppm) = 150.07, 147.99; **IR** (cm^{-1}): ṽ = 2927 (w), 1695 (m), 1593 (m), 1508 (s), 1463 (m), 1363 (w), 1298 (w), 1249 (s), 1177 (s), 1030 (s), 833 (s), 777 (s), 670 (m); **HRMS** (ESI): calcd. for $C_{45}H_{63}N_5O_9PSi^+$ [M+H]$^+$: 876.41272, found: 876.41281.

α-8:
yield: 84%; ***R*f** = 0.40, 0.41 (*i*Hex:EtOAc = 2:1); **^{31}P NMR** (162 MHz, Acetone-d_6): δ (ppm) = 150.20, 148.93; **IR** (cm^{-1}): ṽ = 2926 (m), 1694 (m), 1590 (m), 1508 (s), 1464 (m), 1364 (w), 1230 (w), 1249 (s), 1176 (m), 1031 (s), 978 (m), 833 (s), 778 (s), 701 (m); **HRMS** (ESI): calcd. for $C_{45}H_{63}N_5O_9PSi^+$ [M+H]$^+$: 876.41272, found: 876.41341.

5'-*O*-(4,4'-dimethoxytrityl)-3'-*O*-[2-cyanoethoxy(diisopropylamino)phosphino]-2'-*O*-*tert*-butyldimethylsilyl)-4-*N*-benzoyl-α-cytidine (SI-2)

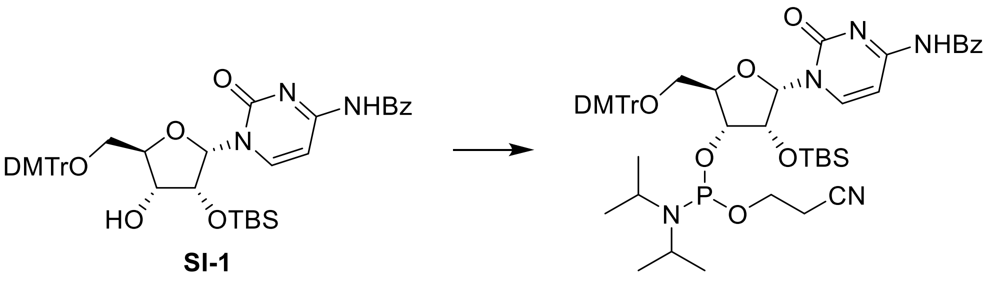

5'-O-(4,4'-dimethoxytrityl-2'-O-tert-butyldimethylsilyl-4-*N*-benzoyl-α-cytidine (**SI-1**) was synthesized in an eight-step procedure according to literature.[2-3] **SI-1** (170 mg, 223 µmol, 1.00 eq.) was pre-dried by lyophilization from benzene and dissolved in DCM (3 mL) at 0 °C under argon atmosphere. DIPEA (155 µL, 890 µmol, 4.00 eq.) and 2-cyanoethyl *N,N*-diisopropylchlorophosphoramidite (124 µL, 556 µmol, 2.50 eq.) were added dropwise. The resulting solution was stirred at room temperature for 5 h and subsequently quenched by the addition of sat. $NaHCO_3$ solution (5 mL). The aqueous layer was extracted with DCM (3 x 10 mL). The combined organic layers were washed with brine (10 mL), dried over Na_2SO_4, and concentrated *in vacuo*. The residue was purified by flash column chromatography (silica, *i*Hex:EE = 2:1 → 1:2 + 0.1% pyridine, HPLC grade solvents) to yield **SI-2** (180 mg, 186 µmol, 84%) as a mixture of diastereoisomers, as a pale-yellow foam.

***R*f** = 0.50, 0.52 (*i*Hex:EtOAc = 1:2); **^{31}P NMR** (162 MHz, Acetone-d_6): δ (ppm) = 150.16, 150.02; **IR** (cm^{-1}): ṽ = 2929 (w), 1667 (m), 1622 (m), 1554 (m), 1508 (m), 1483 (s), 1395 (m), 1299 (m), 1249 (s), 1177 (s), 1157 (m), 1069 (w), 1031 (s), 978 (m), 927 (w), 829 (s), 779 (s), 704 (s); **HRMS** (ESI): calcd. for $C_{52}H_{66}N_5O_9PSi^+$ [M+H]$^+$: 964.44402, found: 964.44278.

Synthesis and purification of oligonucleotides

Phosphoramidites of canonical ribonucleosides (Bz-A-CE, Dmf-G-CE, Ac-C-CE and U-CE) were purchased from LinkTech and Sigma-Aldrich. Oligonucleotides were synthesized on a 1 µmol scale using RNA SynBase™ CPG 1000/110 as solid supports using an RNA automated synthesizer (Applied Biosystems 394 DNA/RNA Synthesizer) with a standard phosphoramidite chemistry. Oligonucleotides were synthesized in DMT-OFF mode using DCA as a deblocking agent in CH_2Cl_2, Activator 42® as activator in MeCN, Ac_2O as capping reagent in pyridine/THF and I_2 as oxidizer in pyridine/H_2O. The cleavage and deprotection of the CPG bound oligonucleotides were performed with a 1:1 aqueous solution mixture (0.6 mL) of 30% NH_4OH and 40% $MeNH_2$. The suspension was heated at 65ºC for 5 min for SynBase™ CPG 1000/110. Subsequently, the supernatant was collected, and the beads were washed with water (2×0.5 mL). The combined aqueous solutions were concentrated under reduced pressure using a SpeedVac concentrator. After that, the crude was dissolved in DMSO (100 µL) and triethylamine trihydrofluoride (125 µL) was added. The solution was heated at 65 ºC for 1.5 h. Finally, the Oligonucleotides were precipitated by adding 3 M NaOAc in water (25 µL) and *n*-BuOH (1 mL). The mixture was kept at -80 ºC for 2 h and centrifuged at 4ºC for 1 h. The supernatant was removed, and the white precipitate was lyophilized. The oligonucleotides were further purified by semi-preparative reverse-phase HPLC using a 1260 Infinity II Manual Preparative LC System from Agilent (G7114A detector) equipped with the column VP 250/10 Nucleodur 100-5 C18ec from Macherey Nagel. A flow rate of 5 mL/min with varying gradients between 0-15% and 0-40% of buffer B in 45 min was applied for the purifications. The following buffer system was used: buffer A: 100 mM NEt_3/HOAc (pH 7.0) in H_2O and buffer B: 100 mM NEt_3/HOAc in 80% (v/v) acetonitrile. The purified oligonucleotides were analyzed by analytical RP-HPLC on a 1260 Infinity II LC System from Agilent (G7165A detector) equipped with the column an EC 250/4 Nucleodur 100-3 C18ec from Macherey Nagel using a flow of 1 mL/min, a gradient of 0-15% or 0-20% of buffer B in 45 min was applied. Finally, the purified oligonucleotides were desalted using a C18 RP-cartridge from Waters. The absorbance of the synthesized oligonucleotides in H_2O solution were measured using an IMPLEN NanoPhotometer® N60/N50 at 260 nm. The extinction coefficients of the oligonucleotides were calculated using the OligoAnalyzer Version 3.0 from Integrated DNA Technologies. For strands containing mainly Isoxazole, extinctions coefficients were calculated based on the base composition method at 223 nm using estimated extinctions coefficients (e.g., 10726 $M^{-1}cm^{-1}$ for IO3 and 7616 $M^{-1}cm^{-1}$ for cytidine). The structural integrity of the synthesized oligonucleotides was analyzed by MALDI-TOF mass measurement. For this purpose, the synthesized oligonucleotides (2-3 µL) were desalted on a 0.025 µm VSWP filter (Millipore), co-crystallized in a 3-hydroxypicolinic acid matrix (HPA, 1 µL) and measured on a Bruker Autoflex II. UV spectra, melting profiles and the concentrations of purified oligonucleotides were measured on a JASCO V-650 spectrometer.

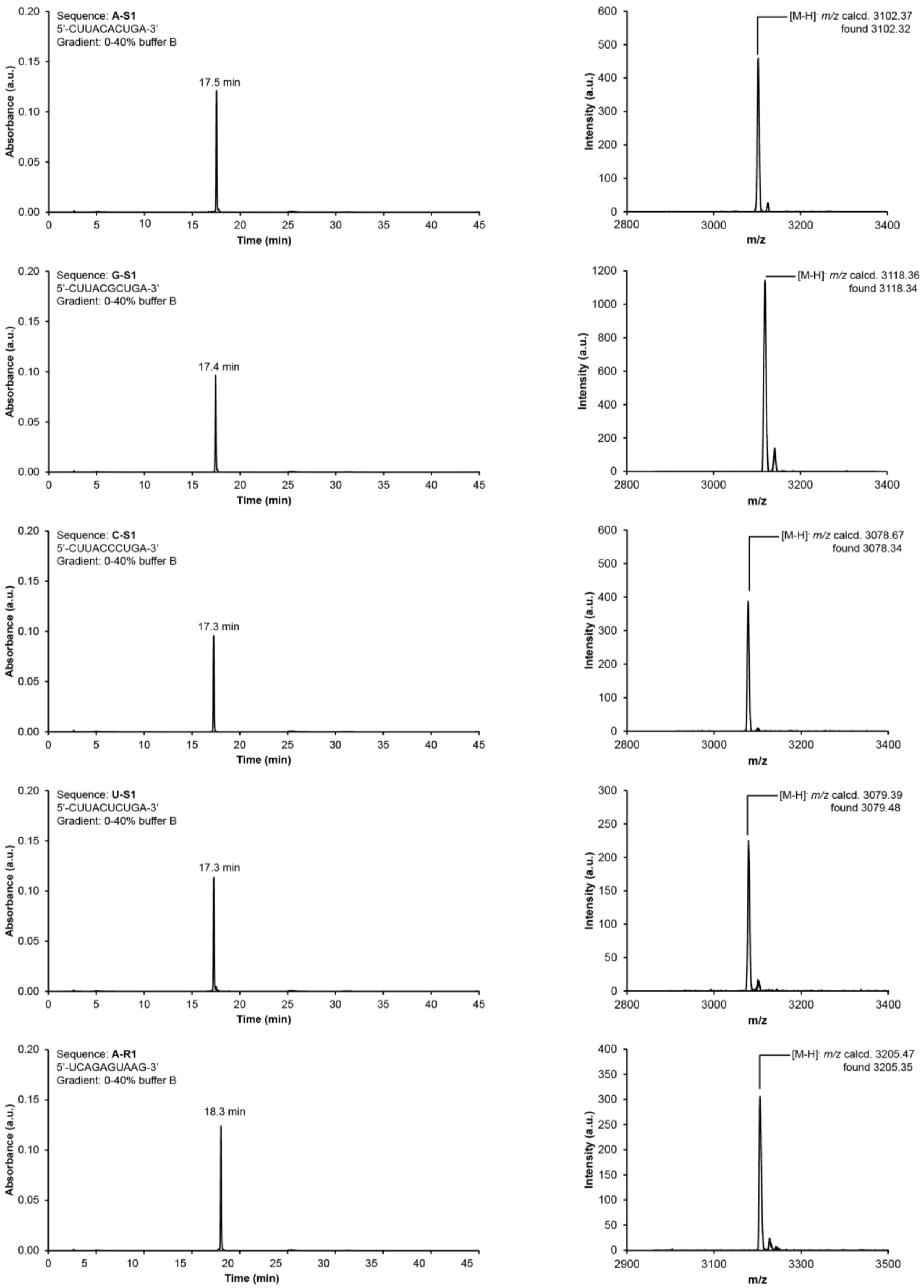

Figure S1. left) HPL-chromatograms and right) MALDI-TOF mass spectra (negative mode) of the purified Oligonucleotides

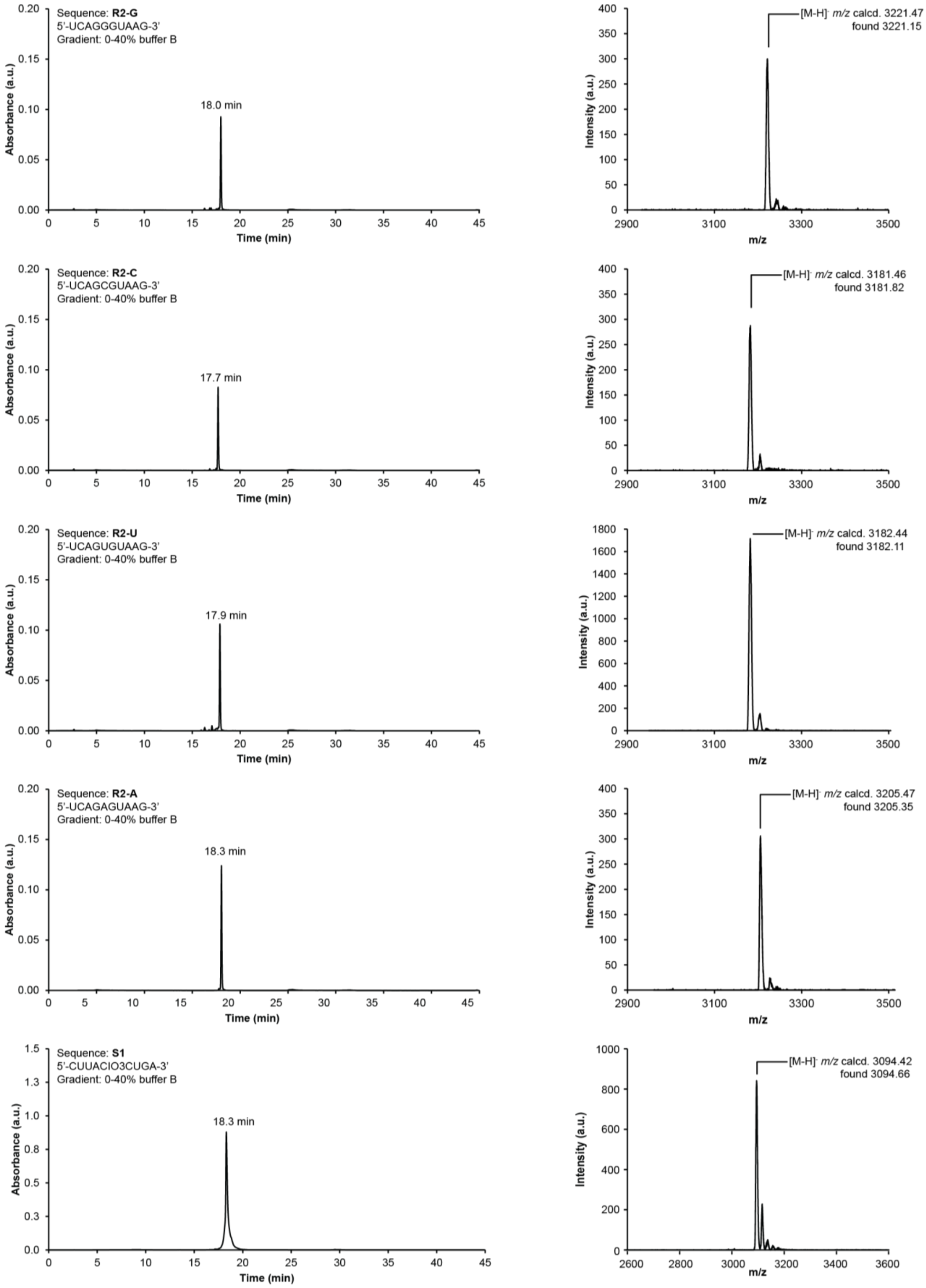

Figure S2. left) HPL-chromatograms and right) MALDI-TOF mass spectra (negative mode) of the purified Oligonucleotides (continued)

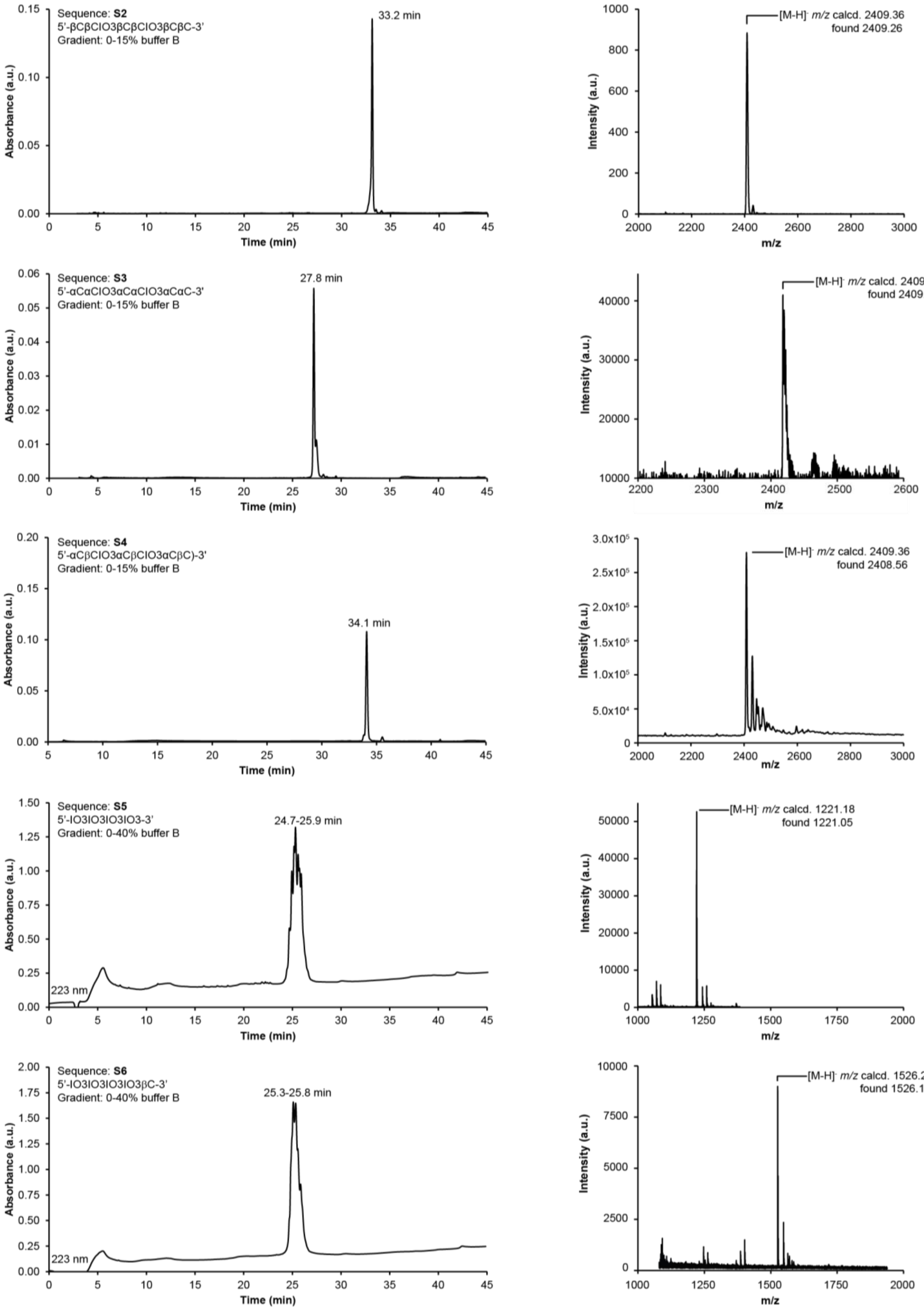

Figure S3. left) HPL-chromatograms and right) MALDI-TOF mass spectra (negative mode) of the purified Oligonucleotides (continued)

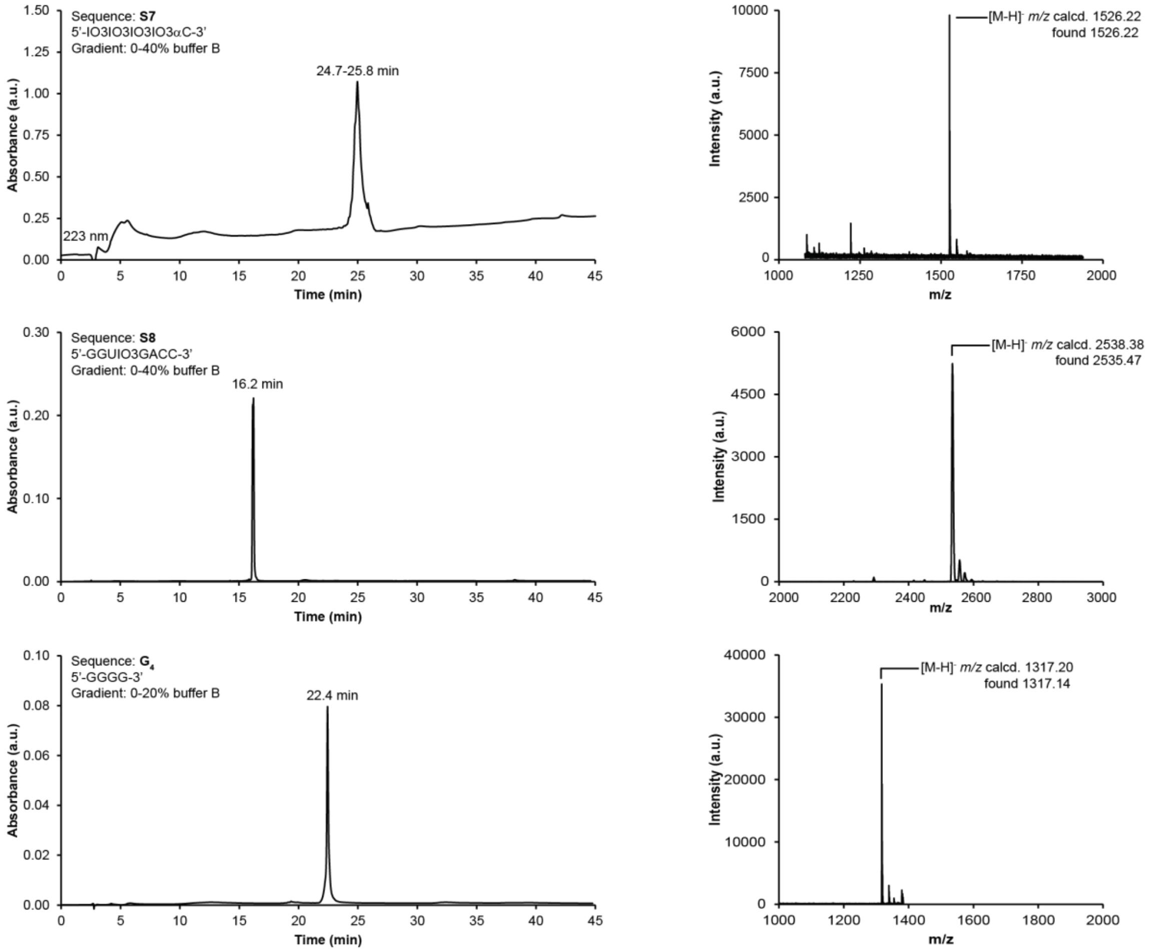

Figure S4. left) HPL-chromatograms and right) MALDI-TOF mass spectra (negative mode) of the purified Oligonucleotides (continued)

UV Melting Curve Measurements

The UV melting curves were measured on JASCO V-650 spectrometer using 10 mm QS cuvettes, purchased from Hellma Analytics. A solution (80 µL) of equimolar amounts of oligonucleotides **S1** and **R1** (4 µM each) in the buffer solution containing 10 mM sodium phosphate buffer (pH 7.0) and 150 mM NaCl was heated at 50°C for 5 min and gradually cooled to 4°C prior to the measurement. Melting profiles were recorded at temperatures between 5 and 70°C with a ramping and scanning rate of 1°C/min at 260 nm. All samples were measured at least three times.

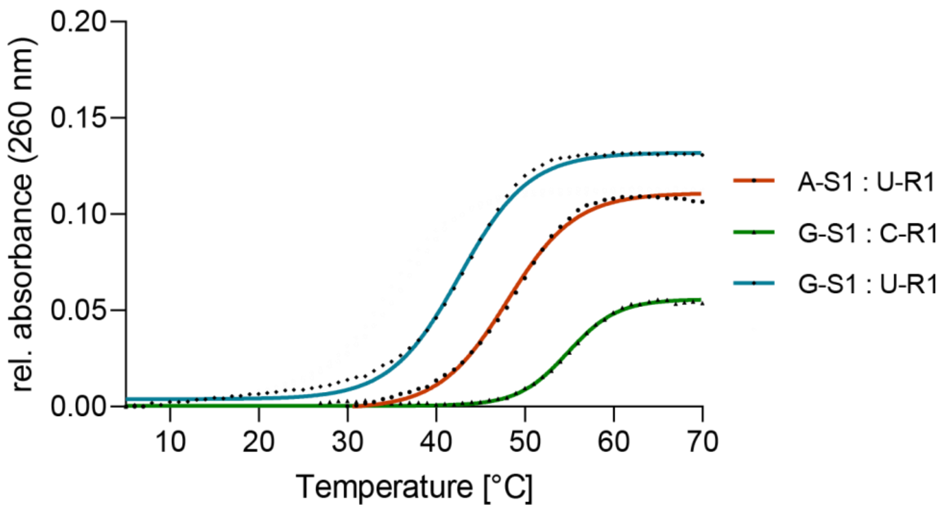

Figure S5. UV Melting curves of dsRNAs: **A-S1:U-R1** (T_m = 48.0°C), **G-S1:C-R1** (T_m = 54.8°C), **G-S1:U-R1** (T_m = 42.6°C). Line shows the fit of the data to a two-state melting model using a mono-sigmoidal Boltzmann function.

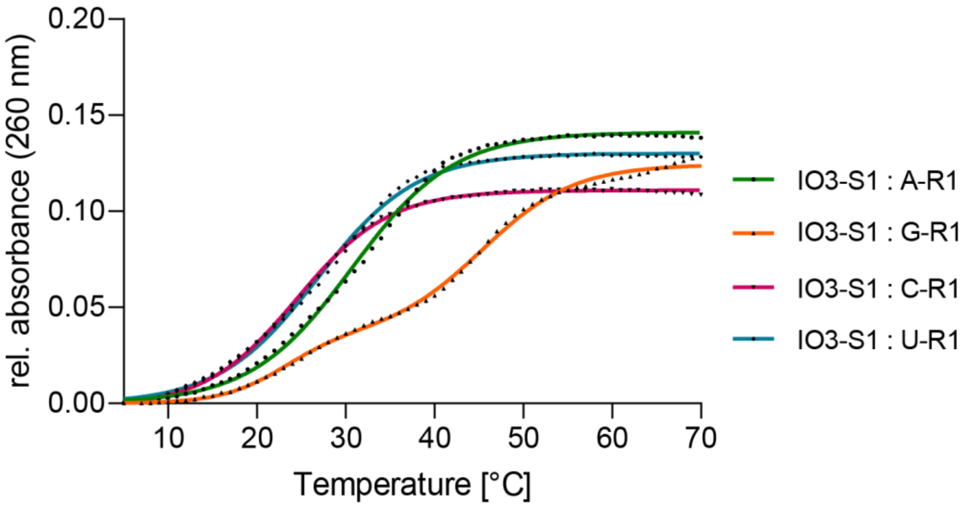

Figure S6. UV Melting curves of dsRNAs: **IO3-S1:A-R1** (T_m = 30.7°C), **IO3-S1:G-R1** (T_m = 44.6°C), **IO3-S1:C-R1** (T_m = 24.5°C),), **IO3-S1:C-R1** (T_m = 26.8°C). Line shows the fit of the data to a two-state melting model using a mono-sigmoidal Boltzmann function.

In-strand cytidine formation reactions

Stock solutions of boric acid (pH 9.7, 100 mM) and Na_2CO_3 (1000 mM) were prepared in water. The oligonucleotide **S1**-**S7** (2-10 nmol) was with mixed with buffer, Na_2CO_3, and water. The Fe^{2+} source (FeS or FeS_2) and DTT were added to the mixture. The final concentration of the components: 100 uM of Oligo, 50 mM Buffer, 100 mM Na_2CO_3, 100 mM Fe^{2+}, 300 mM DTT. The mixture was heated at 90 °C for 2 h in a *TAdvanced Thermocycler* by *Biometra*. After cooling to room temperature, the solids were removed by centrifugation and washed with water (2×0.2 mL), residual solids were removed using a syringe filter (0.20 um, PTFE-membrane). The reaction mixture was concentrated by lyophilization and subsequently analyzed by reverse-phase HPLC. The yields of the reactions were calculated by integration of the chromatographic peaks of the products and the use of the calibration curves of the synthetically prepared product.

Digestion and LC-HESI-MS analysis

Reaction buffer 10X and *Enzyme mix* was bought as a Nucleoside Digestion Mix (M0649S) kit (*New England BioLabs Inc*.). The purified oligonucleotide (250-500 ng in 46 uL) was incubated with *Reaction buffer 10X* (5 uL) and *Enzyme mix* (1 uL) at 37°C for 1.5 h. The mixture was subsequently diluted to 90 uL and then analyzed by LC-HESI-MS on a Thermo Finnigan LTQ Orbitrap XL and were chromatographed by a Dionex Ultimate 3000 HPLC system. All chromatographic separations except for nucleotides were performed on an Interchim YMC-Triart C18 column column with a flow of 0.15 ml/min and a constant column temperature of 30 °C The following buffer system was used: buffer A: 2 mM $HCOONH_4$ in H_2O (pH 5.5) and buffer B: 2 mM $HCOONH_4$ in 80% (v/v) acetonitrile (pH 5.5). The elution was monitored at 223 nm and 260 nm (Dionex Ultimate 3000 Diode Array Detector). The chromatographic eluent was directly injected into the ion source without prior splitting. Ions were scanned by use of a positive polarity mode over a full-scan range of m/z 80-500 with a resolution of 30000. Nucleotides were scanned by use of a negative polarity mode over a full-scan range of m/z 120-1000 with a resolution of 30000. The synthetic standards for the co-injection experiments were synthesized in our lab (see synthetic procedures or according to reported literature[3]) or purchased.

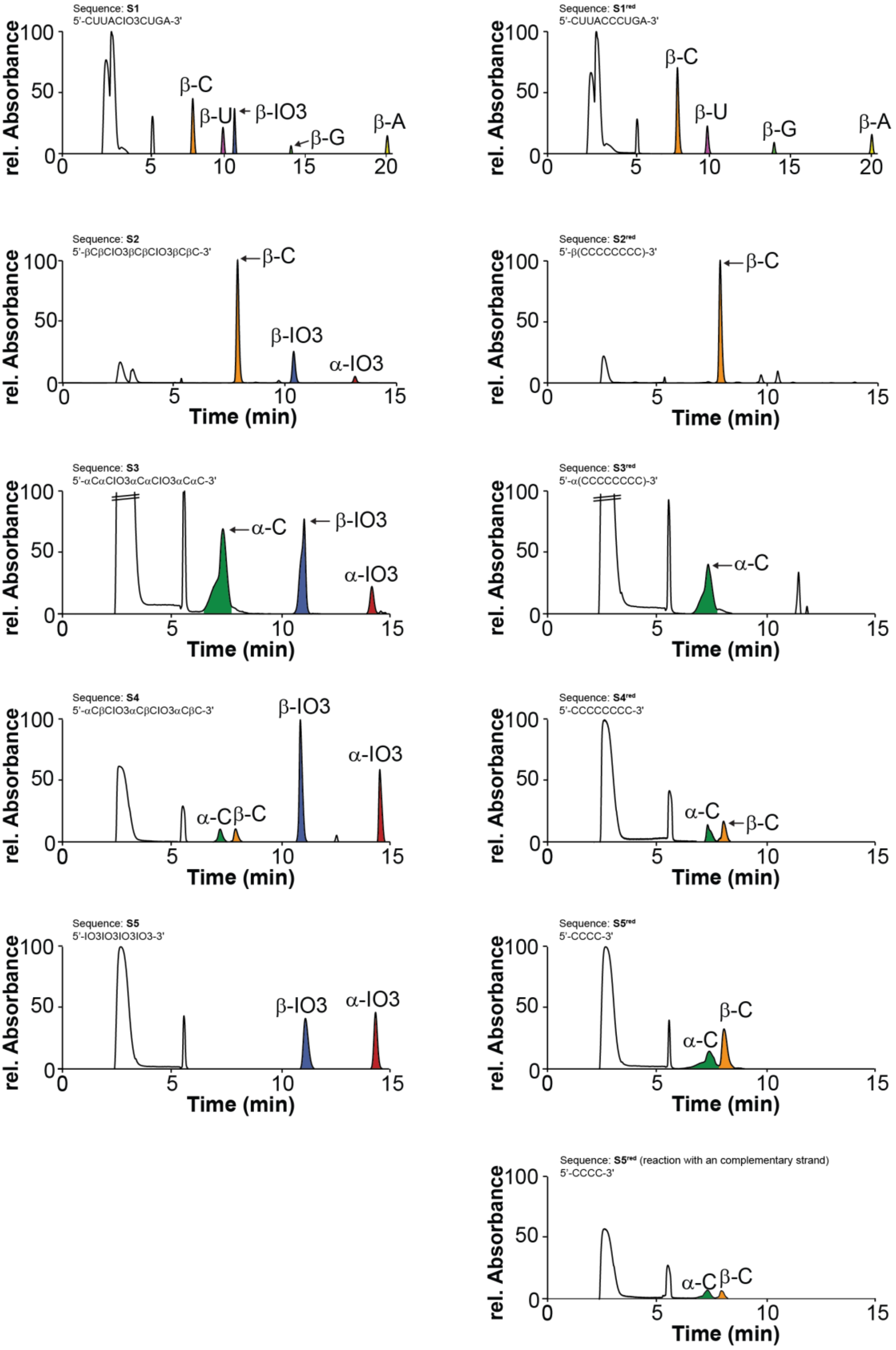

Figure S7. HPLC-MS chromatograms of strands **S1-S7** (left) and their respective N-O cleavage and cyclization products **S1**^{red}**-S7**^{red} (right).

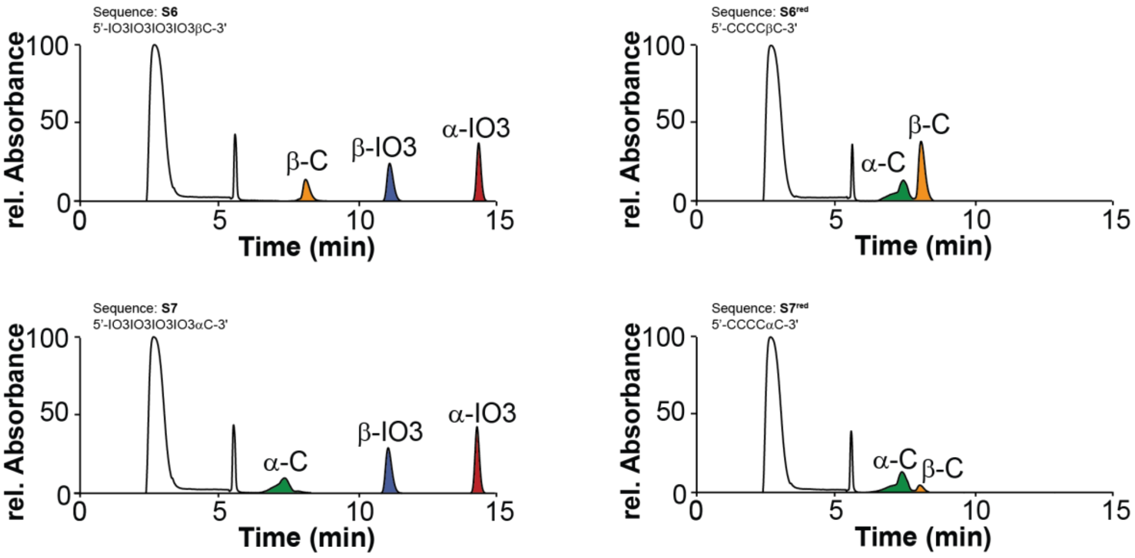

Figure S8. HPLC-MS chromatograms of strands **S1-S7** (left) and their respective N-O cleavage and cyclization products **S1^{red}-S7^{red}** (right).(continued)

High-resolution NMR studies of IO-containing RNA oligonucleotides

Canonical dsRNA sample **Can_8** (Figure S1) was purchased after purification via high-performance liquid chromatography (HPLC) from Ella Biotech GmbH (Martinsried, Germany). The modified dsRNA sample **S7** (Figure S1) was synthesized as described above. We dissolved both Can_8 and S7 samples in an aqueous phosphate buffer. The buffer consisted in Na_2HPO_4/NaH_2PO_4 (15 mM, pH 7.0) and NaCl (25 mM) in H_2O. We added sodium trimethylsilylpropanesulfonate (DSS) (50 µm) as a chemical shift standard for proton NMR spectroscopy, NaN_3 (0.01% m/w) to avoid the growth of bacteria in the sample and D_2O (5%, 9.0 µL) as a lock reference for the spectrometer. Both samples were measured in 3 mm NMR tubes, with a final volume of 0.189 mL and a concentration of 0.53 mM and 1.1 mM, respectively. Experiments were performed on a Bruker Avance III spectrometer operating at a ^1H Larmor frequency of 800 MHz equipped with a 5 mm triple channel ^1H, ^{13}C and ^{15}N cryoprobe. Spectra were acquired and processed using Topspin 3.5 and 4.0, respectively, and further analyzed by NMRFAM-Sparky.[4] Unless otherwise stated, all experiments were measured at 2 °C to ensure the presence of a single conformer in solution. ^1H–^1H NOESY spectra were measured with mixing time points at 40, 80, 120, 160, 200, 240, 280, and 320 ms; with 8192 x 1024 complex points, 234 and 29.3 ms acquisition times in the direct and indirect dimension, respectively. Water suppression was achieved using excitation sculpting pulse sequences. The experimental time was about 6 hours for each 2D NOESY spectrum. ^1H–^1H TOCSY spectra were recorded with a mixing time of 80 ms using MLEV17 mixing scheme, with 4096 x 256 complex points, 51 and 16 ms acquisition times in the direct and indirect dimension, respectively. For the purpose of comparison, the same measurements were performed for the canonical and the modified 8-mer. The used methods as well as the conditions for both samples are listed below. For resonance assignment standard ^1H–^1H NOESY spectra were measured as well as a TOCSY for the modified oligomer. For more detailed analysis a natural abundance ^1H–^{15}N HMQC was recorded using the SOFAST pulse sequence and a ^1H–^{13}C HSQC was recorded using a Bruker standard pulse sequence. The basis of the assignment of a NOESY spectra is provided by so-called 'sequential paths', which are formed by NOE cross peaks from adjacent ribose backbone base segments.

Molecular modelling of IO-containing RNA oligonucleotides

For the structure calculation the parameters for the modified base first needed to be incorporated into the topology and the parameter files used by CNSsolve to generate an RNA structure.[5] The bond length, angles as well as dihedral angles were determined using an optimized structure of the modified nucleoside. The calculation for this purpose was performed using a standard Hartree Fock method with a 6-31G base. After the integration of the modified base, the three-dimensional structure was determined by an *in-silico* annealing procedure. The restriction data used as input for the calculation has been directly derived from the ^1H–^1H NOESY spectra. Due to the characteristic of nucleic acids having a limited amount of protons, the data obtained from the ^1H–^1H NOESY alone is not sufficient to provide the software with enough input for a *de novo* structure calculation. For this reason, the sugar-phosphate backbone the dihedral angles were determined according to the literature values for an A-form RNA, which was assumed due to the consistency of the fingerprint region with said conformation. For all canonical bases (meaning all the bases except for IO3) the dihedral angles, bond length and bond angles embedded in the CNSsolve program were used. Besides the ^1H–^1H distances extracted from NOESY experiments, the distances between carbon, oxygen and nitrogen atoms were identified, gauging a 3D model of the canonical structure using Chimera 1.14. The distances were added to the restraint distance file supplementing the NOESY data. Approximate ^1H–^1H distances were defined by the NOESY spectra recorded at different (40–320 ms) mixing times. The distances could be divided into two categories based on cross-peaks intensities. Protons close in space are therefore visible for short mixing times, conversely protons further away only appear at higher mixing times. The extracted peak heights were converted to distances taking the cross-peak between C7H5 and C7H6 as a reference due to the fixed and known distance of 2.421 Å between the two protons. The values were generated according to the relationship that the intensity is reciprocal to the sixth of the distance, $J \sim 1/r^6$.[6] Overlapping signals were excluded from the analysis and did not contribute to the molecular modelling procedure.

Schematic sample representation

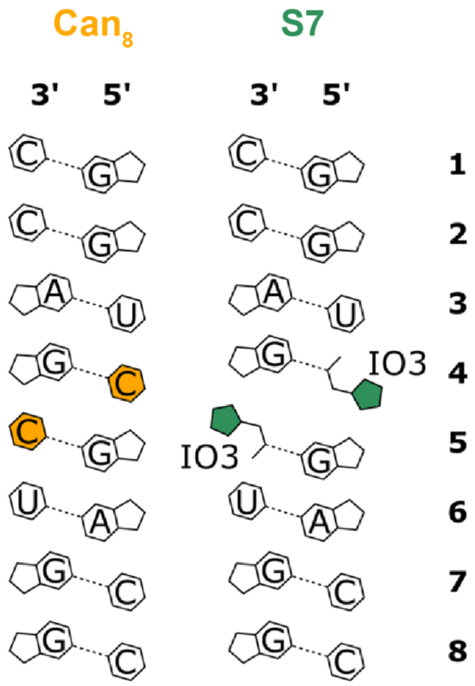

Figure S9. Scheme of the canonical (Can₈, left) and modified (S7, right) RNA samples

Homo- and heteronuclear correlation spectra

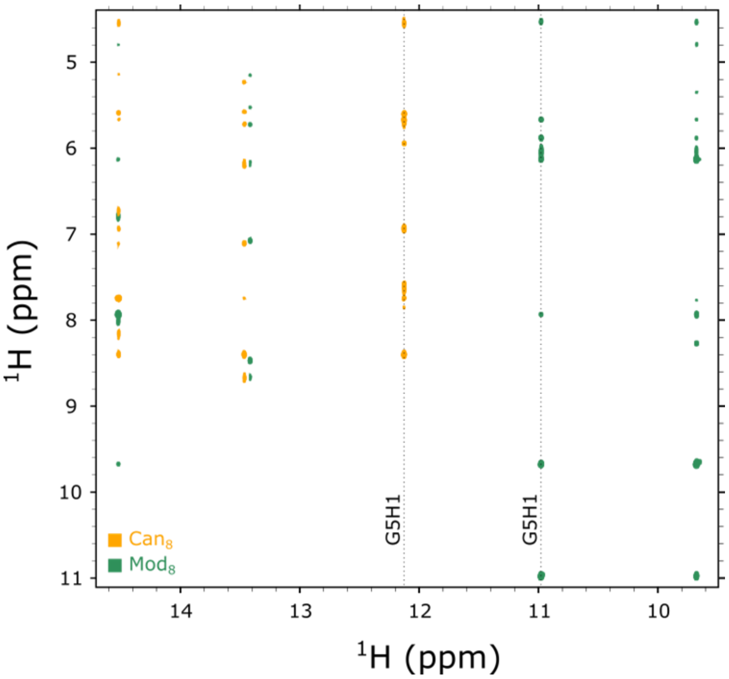

Figure S10. Overlap of the NOESY spectra measured with τ=320 ms for Can₈ and S7 showing the imino region. The chemical shift difference of G5H1, reporting on the stability of the IO3-G base pair, is highlighted by the dotted lines.

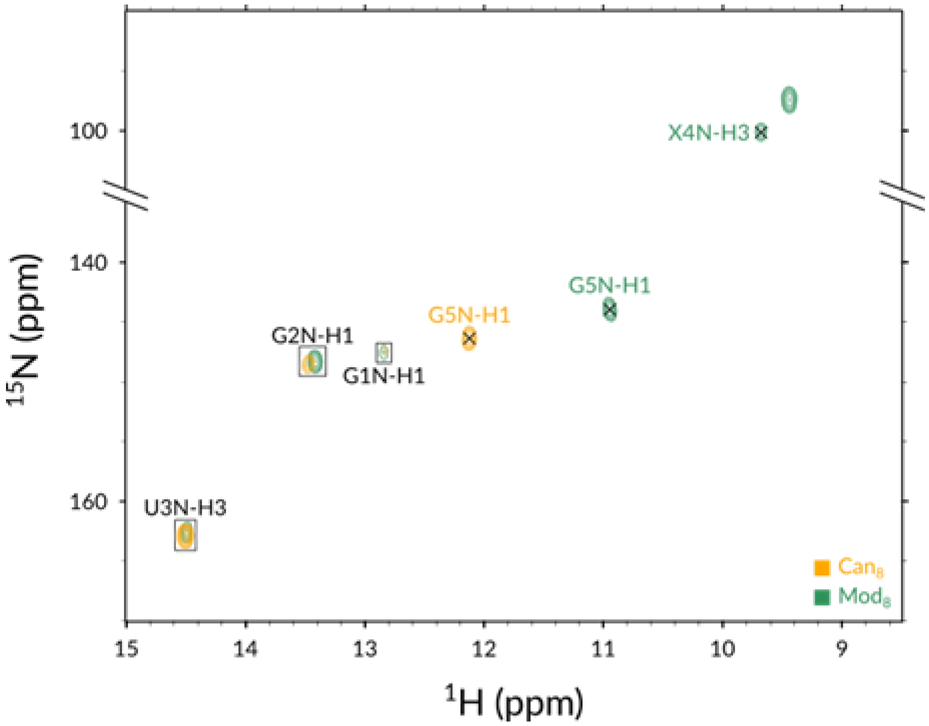

Figure S11. Comparison of the ^{1}H-^{15}N HMQC spectra for Can₈ (yellow) and S7 (green) highlighting the nuclei involved in base pairing

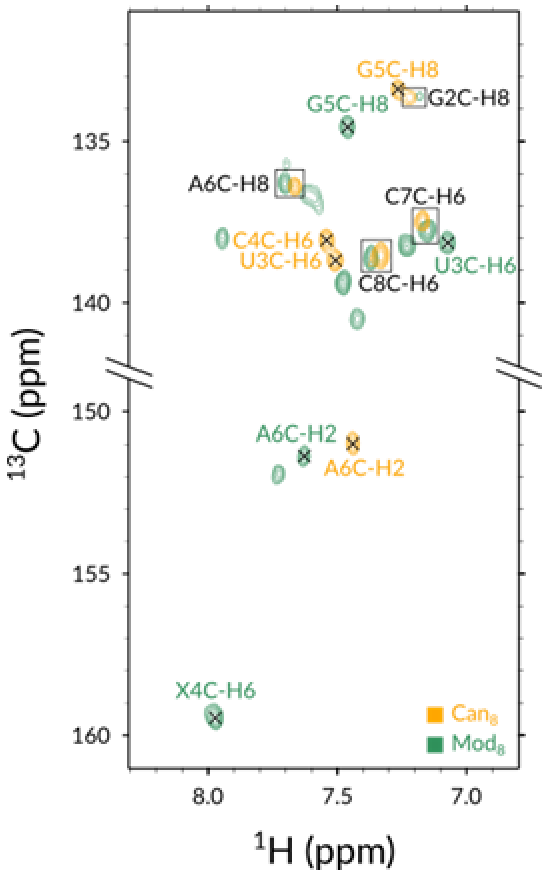

Figure S12. Comparison of the aromatic region of ^{1}H-^{13}C HSQC spectra for Can_8 (yellow) and S7 (green).

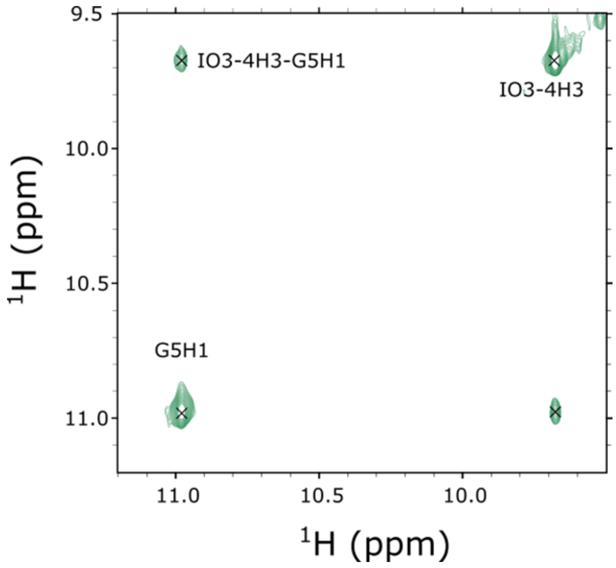

Figure S13. Excerpt from the ^{1}H-^{1}H NOESY spectrum (t_{mix} = 40 ms) of S7 showing the interstrand cross-peak between G5H1 and IO3-4H3.

Chemical shift assignment tables

Table S1. Chemical shifts of the signals for Can_8 and S7

Nucleobase	Nucleus	Can_8 chemical shift	S7 Chemical shift
G2	H1	13.464	13.414
	N1	148.5	148.3
	H1'	5.927	5.912
	H2'	4.533	4.567
	H3'	4.615	4.476
	H5'	4.144	4.151
	H8	7.515	7.551
	C8	133.70	133.61
U3	H1'	5.580	5.529
	H2'	4.549	4.837
	H3	14.517	14.520
	N3	162.9	162.6
	H5	5.140	4.799
	H5'	4.112	4.115
	H6	7.819	7.385
	C6	139.07	138.44
X4	H1'	5.594	5.348
	H2'	4.565	4.539
	H3'	4.459	4.881
	H5'	4.126	3.994
G5	H1	12.126	10.979
	N1	146.3	144.4
	H1'	5.602	5.674
	H3'	4.498	4.525
	H5'	4.132	4.149
	H8	7.577	7.772
	C8	133.78	134.94

Nucleobase	Nucleus	Can_8 chemical shift	S7 Chemical shift
A6	H1'	5.946	5.879
	H2	7.746	7.934
	C2	151.28	151.65
	H2'	4.568	4.531
	H3'	4.682	4.679
	H5'	4.135	4.139
	H8	7.982	8.025
	C8	136.73	136.68
C7	H1'	5.381	5.439
	H2'	4.151	4.210
	H3'	4.360	4.355
	H41	8.400	8.469
	H42	7.109	7.080
	H5	5.231	5.157
	H6	7.481	7.469
	C6	137.84	138.15
C8	H1'	5.722	5.730
	H2'	4.025	4.178
	H41	8.412	8.365
	H42	6.990	7.006
	H5	5.476	5.475
	H5'	3.920	4.038
	H6	7.638	7.674
	C6	138.89	138.97

NMR spectra of synthesized compounds

^{1}H and ^{13}C{^{1}H} NMR spectra of compound 11

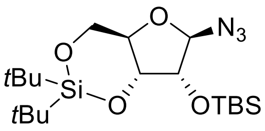

11

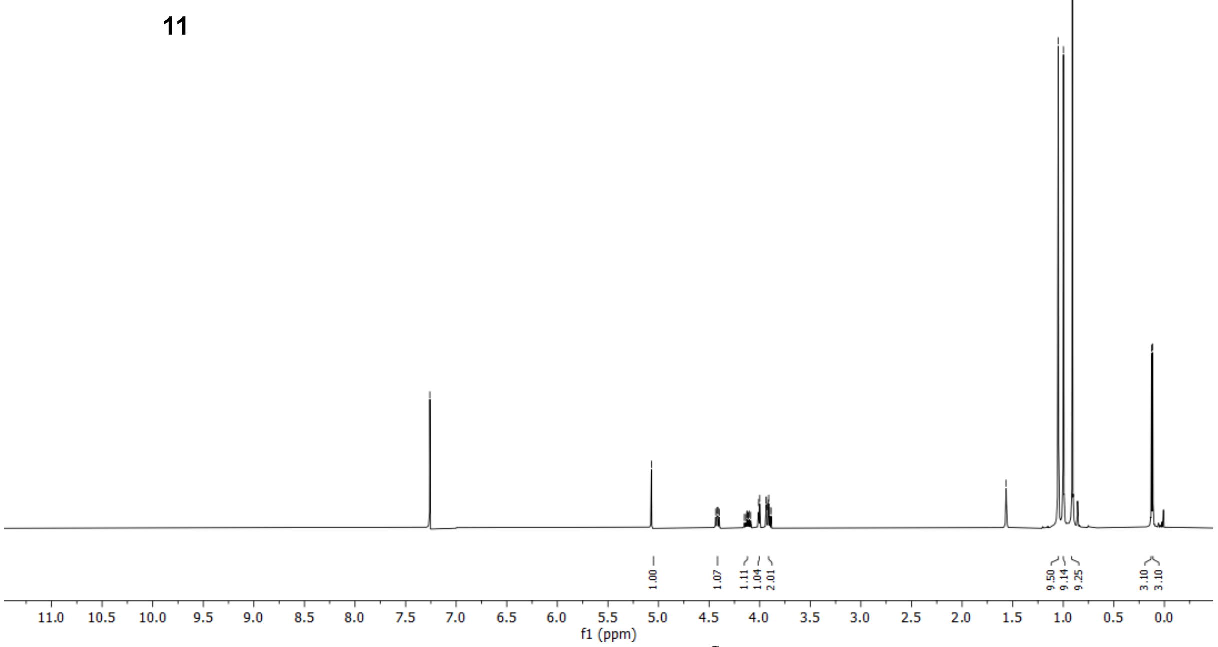

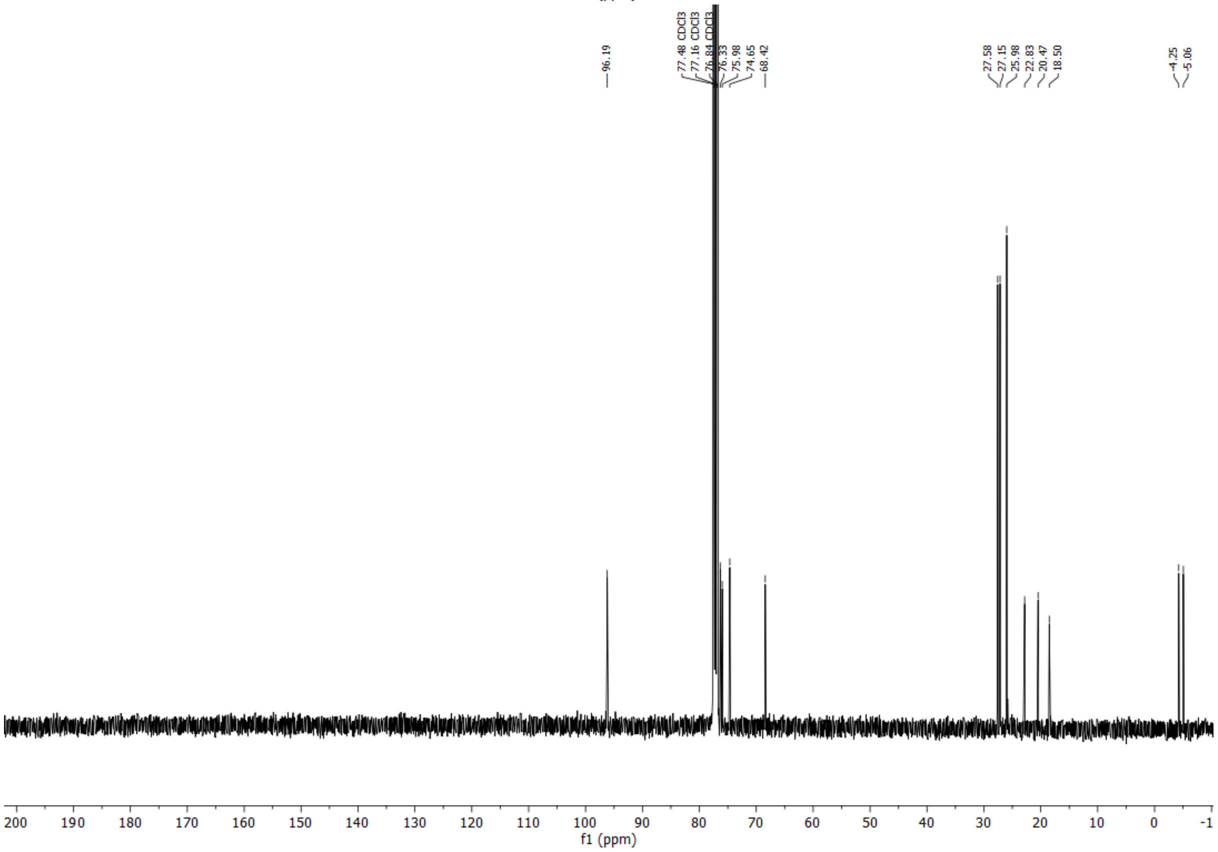

^{1}H and $^{13}C\{^{1}H\}$ NMR spectra of compound α-12

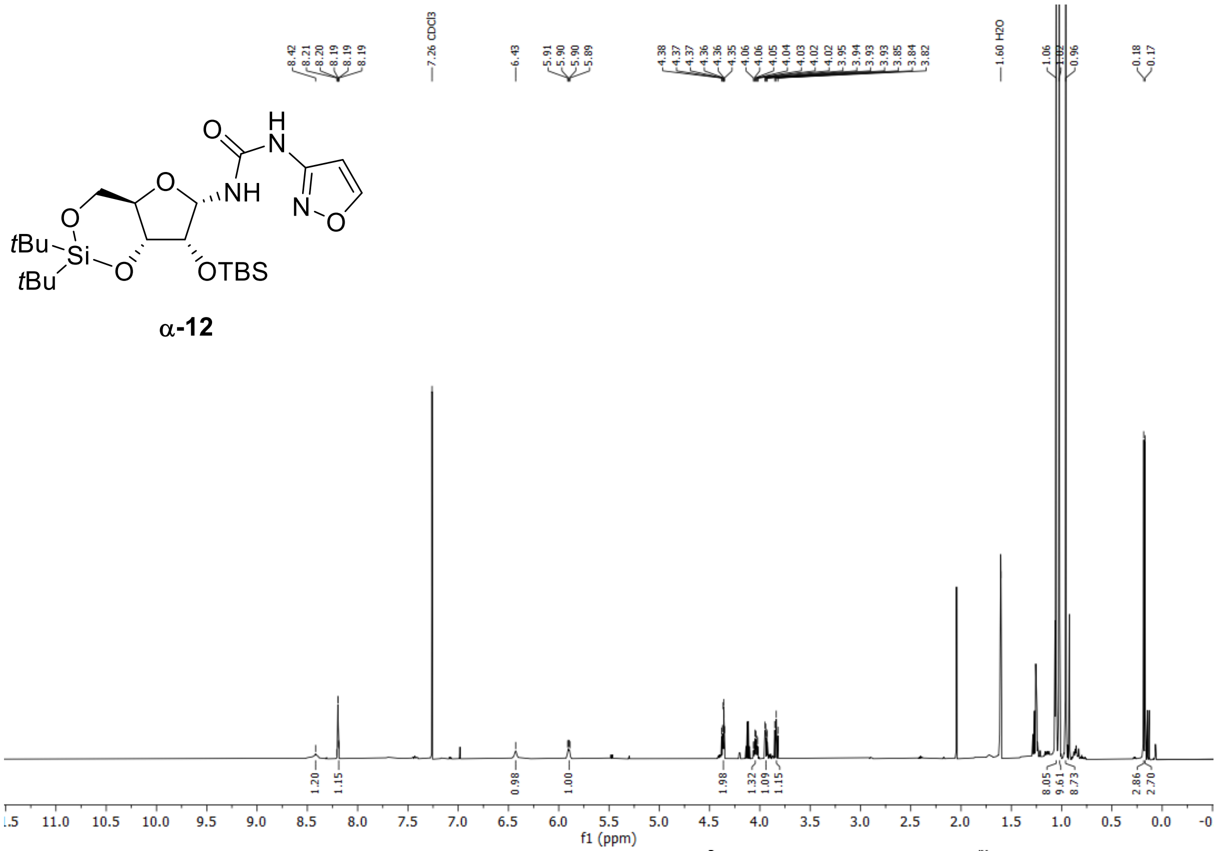

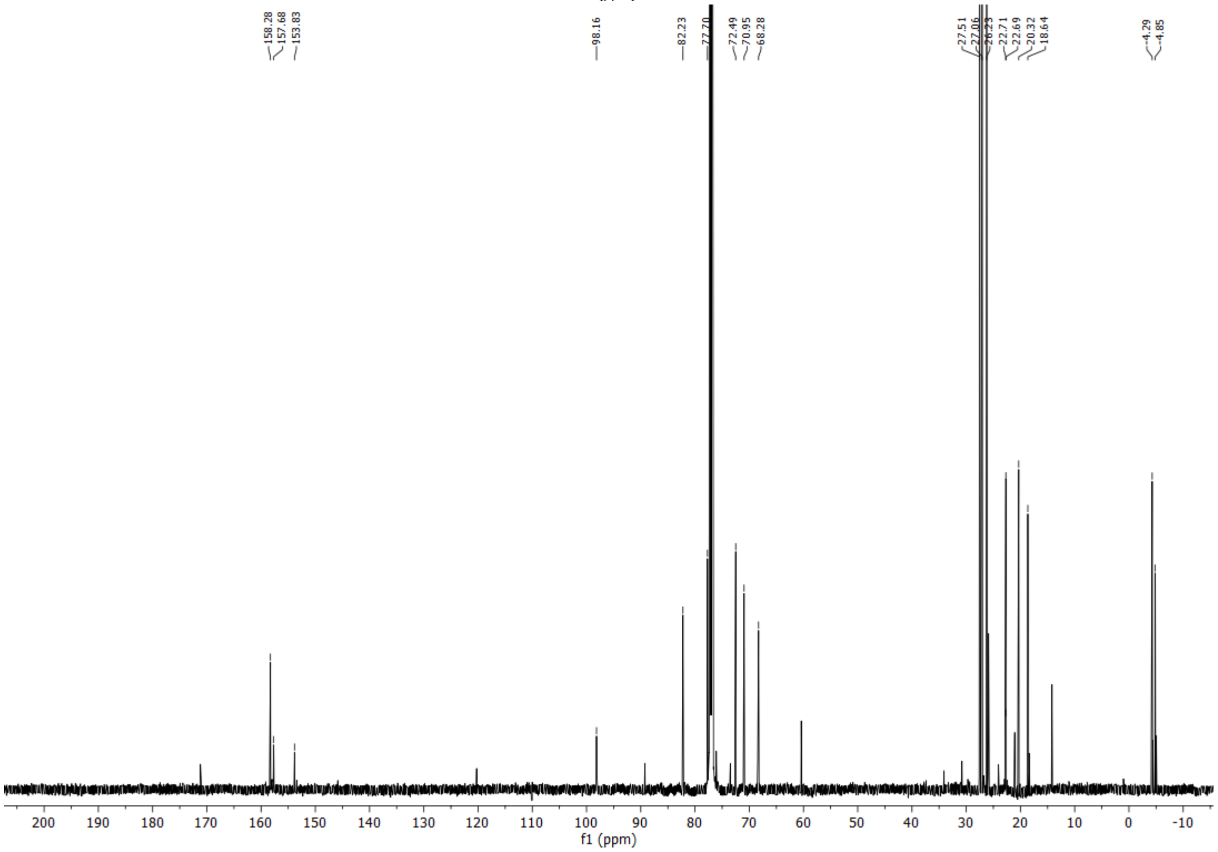

^{1}H and $^{13}C\{^{1}H\}$ NMR spectra of compound β-12

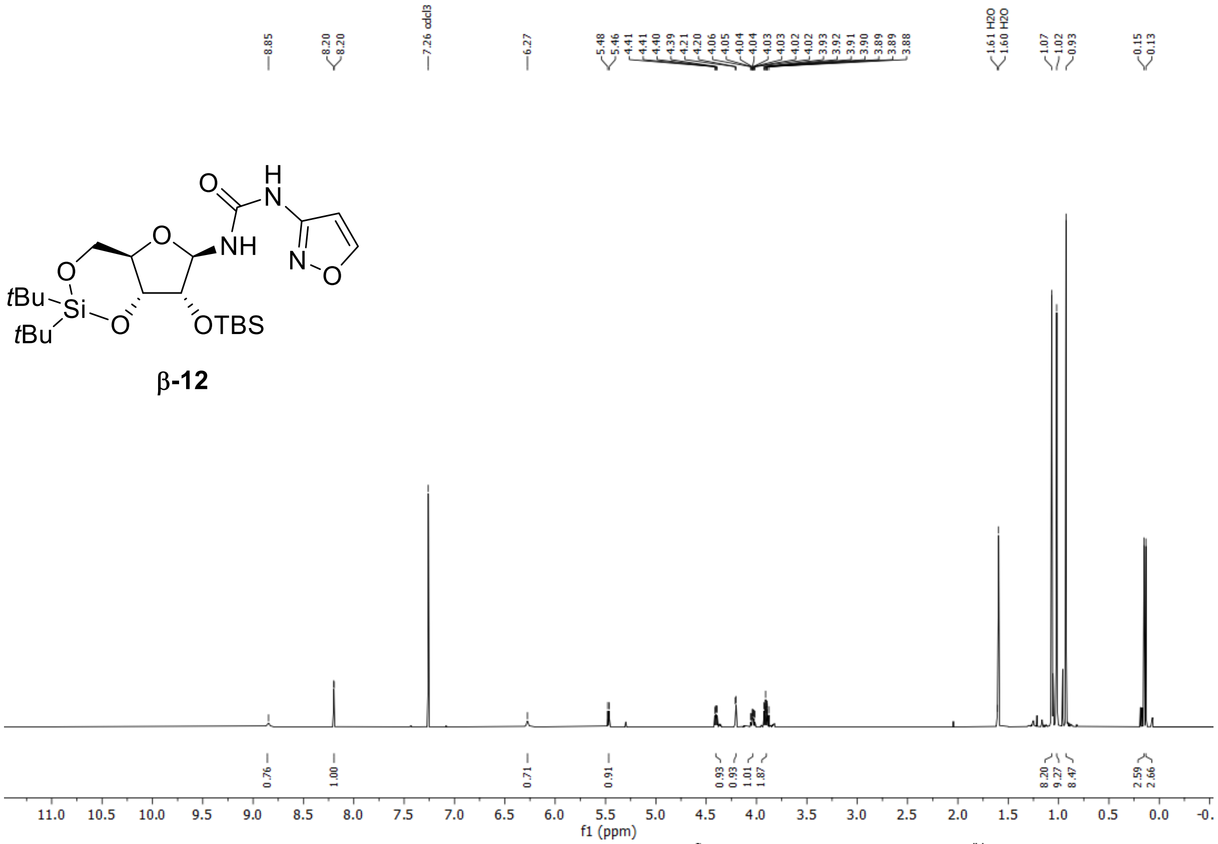

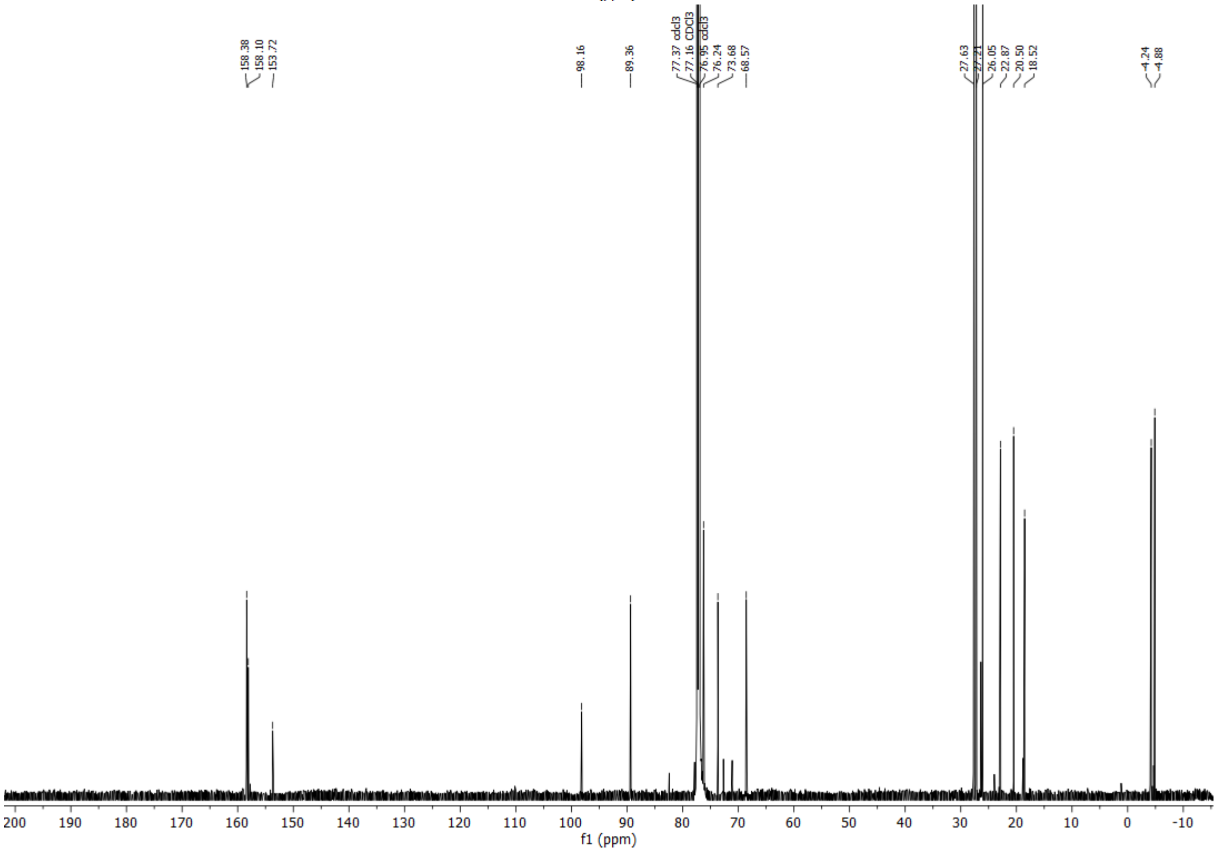

^{1}H and ^{13}C{^{1}H} NMR spectra of compound α-13

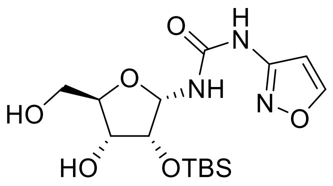

α-13

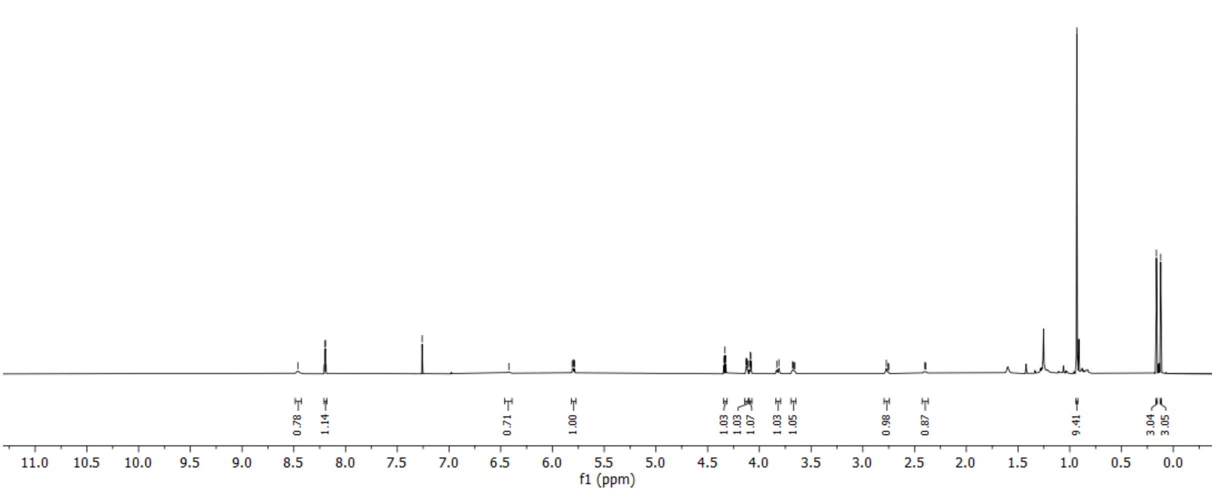

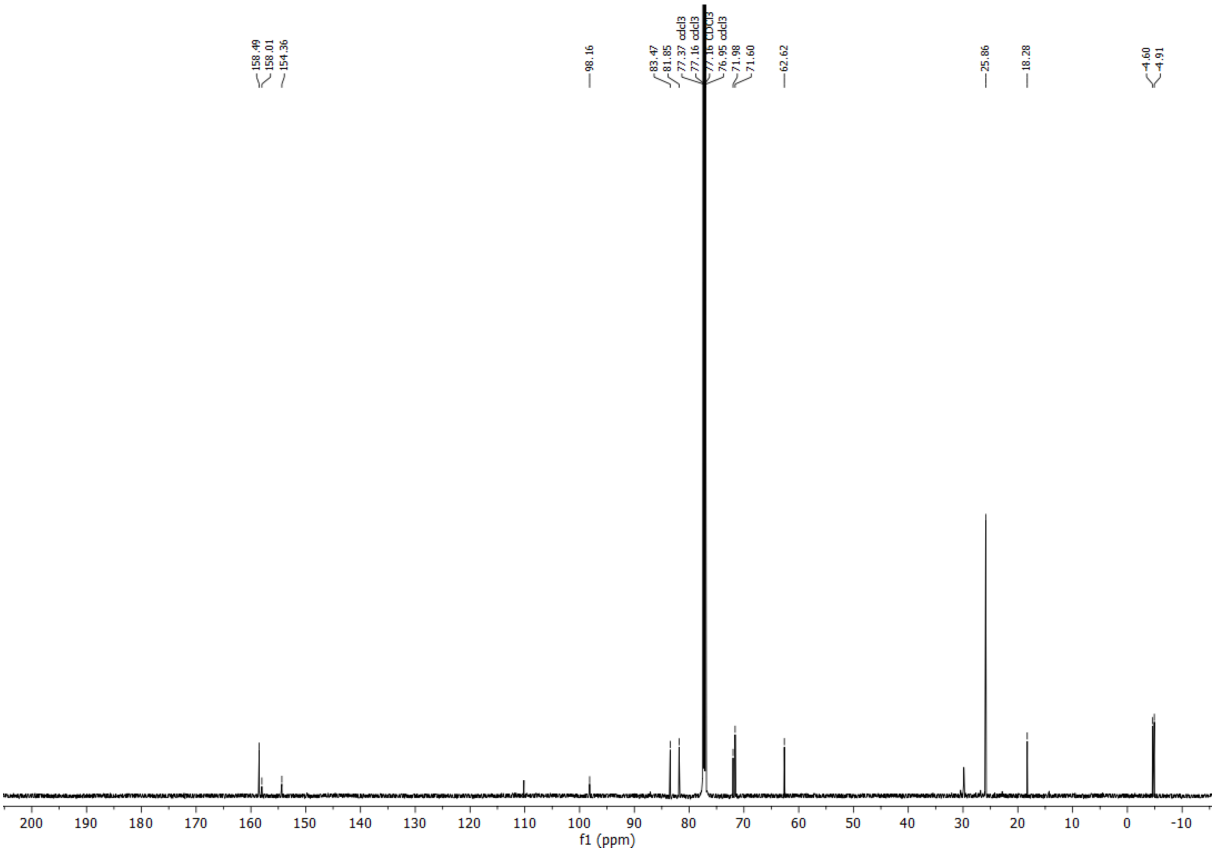

^{1}H and $^{13}C\{^{1}H\}$ NMR spectra of compound β-13

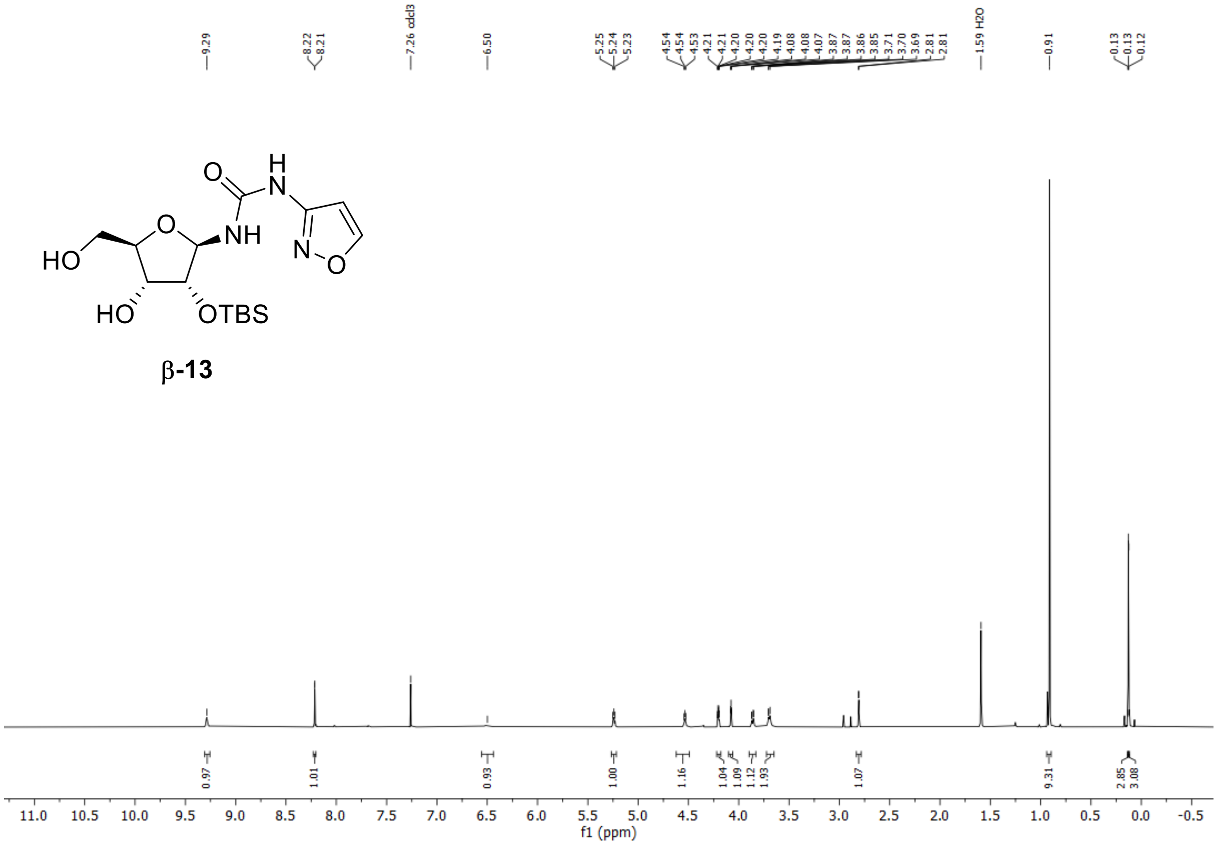

β-13

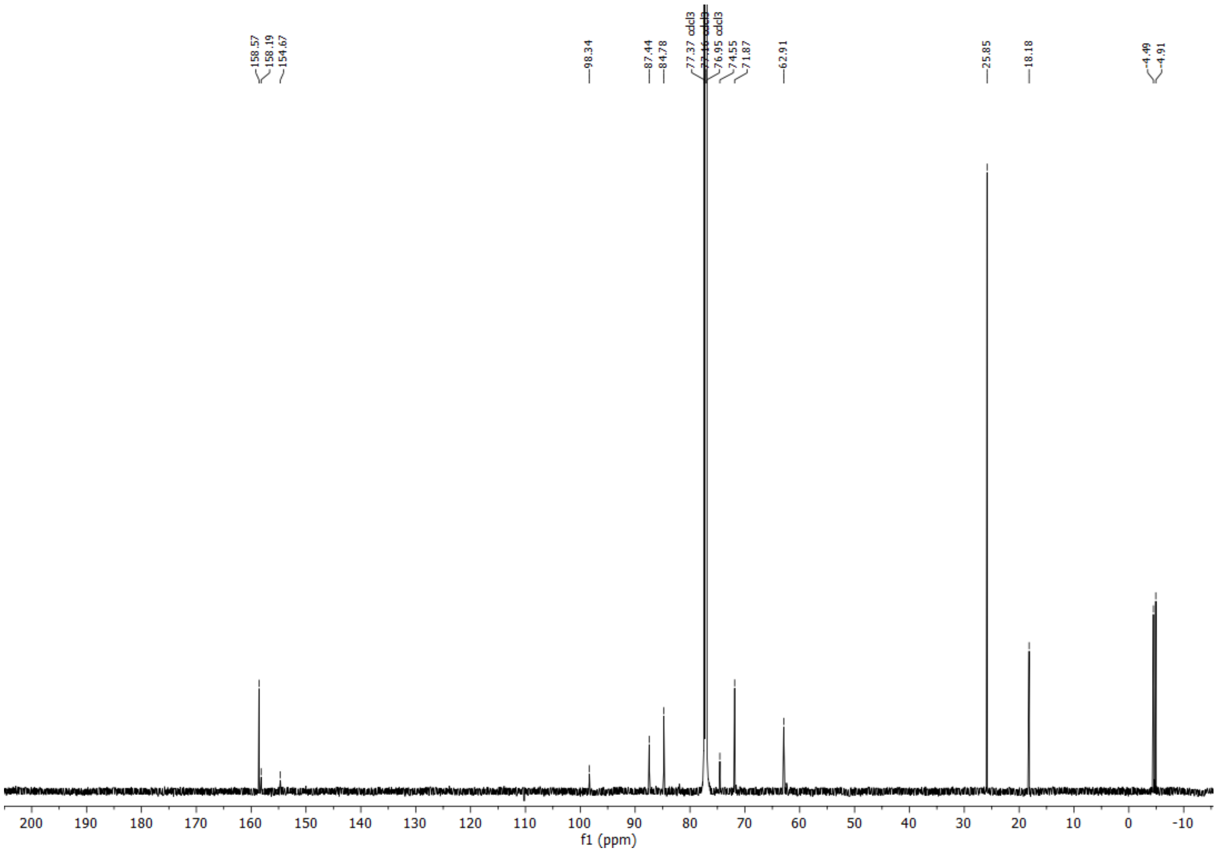

^{1}H and ^{13}C{^{1}H} NMR spectra of compound α-14

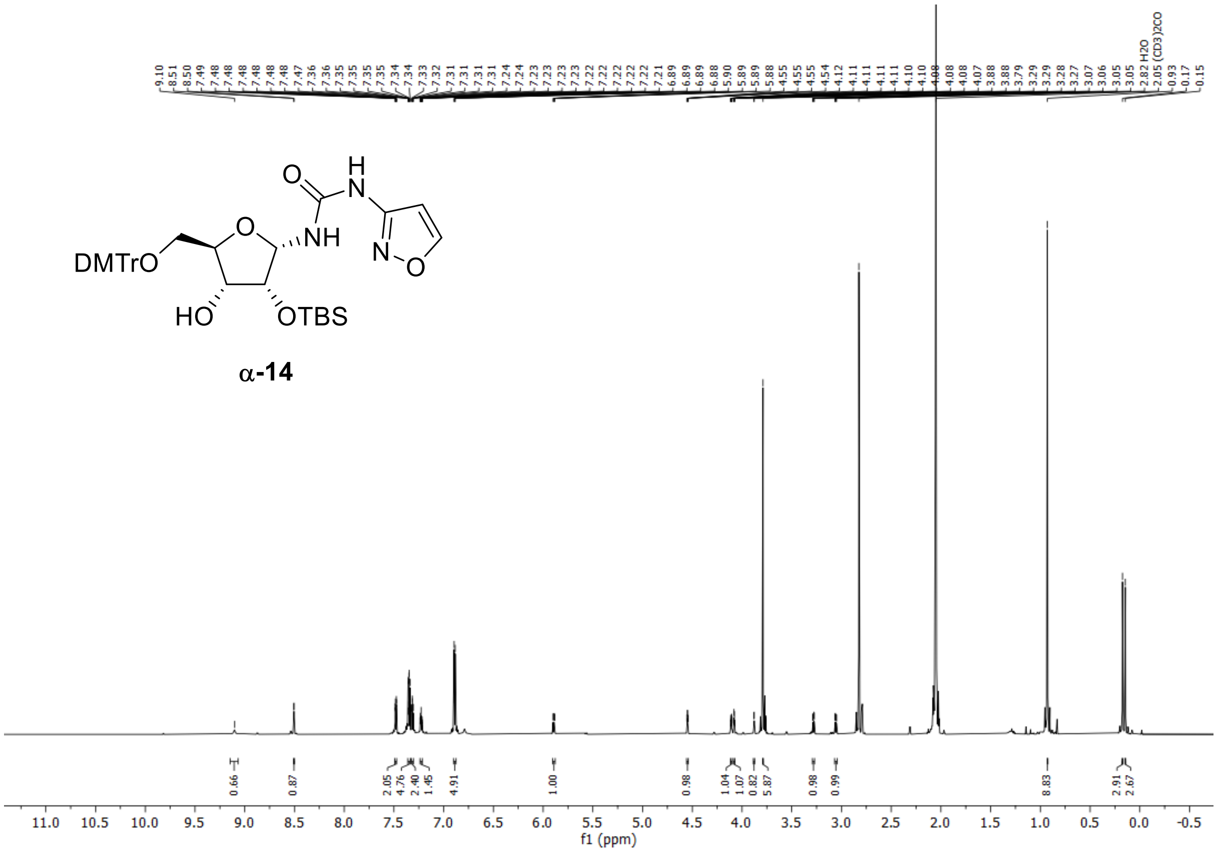

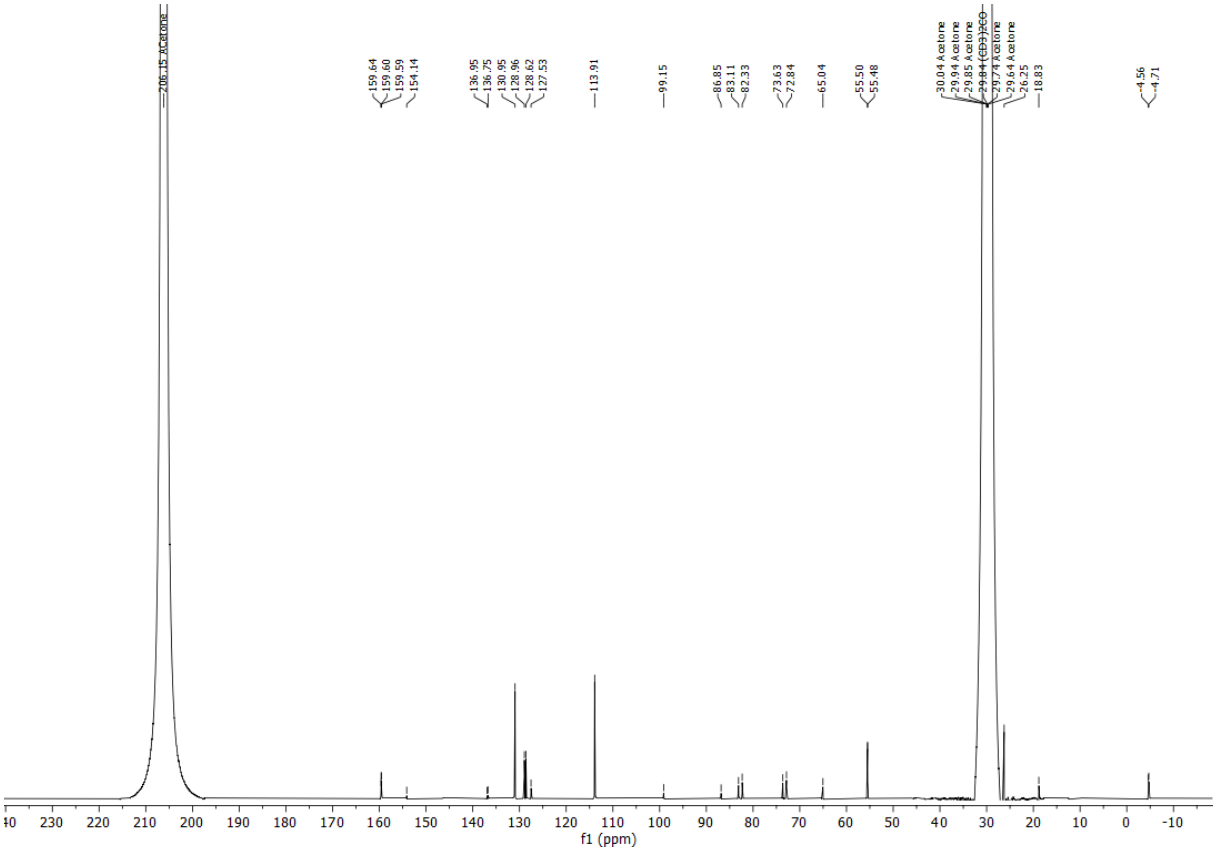

^{1}H and $^{13}C\{^{1}H\}$ NMR spectra of compound β-14

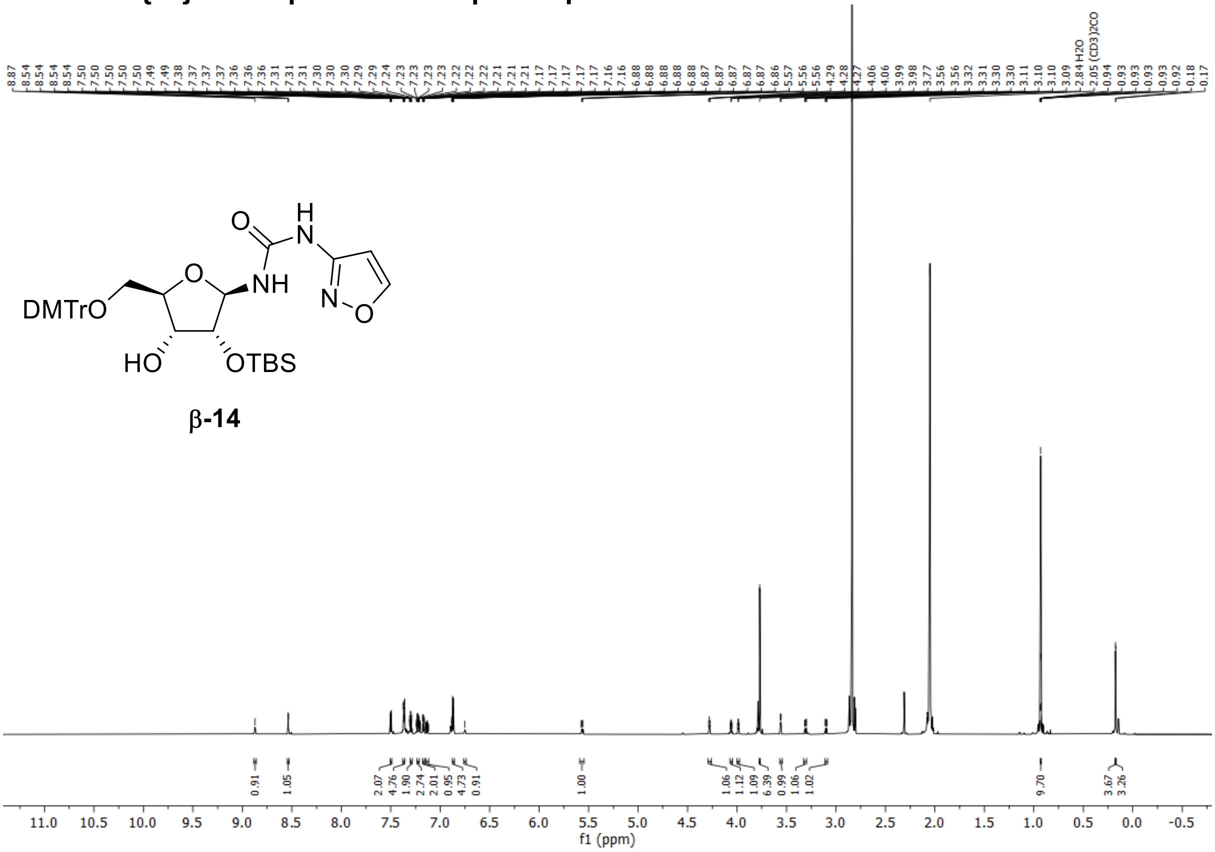

β-14

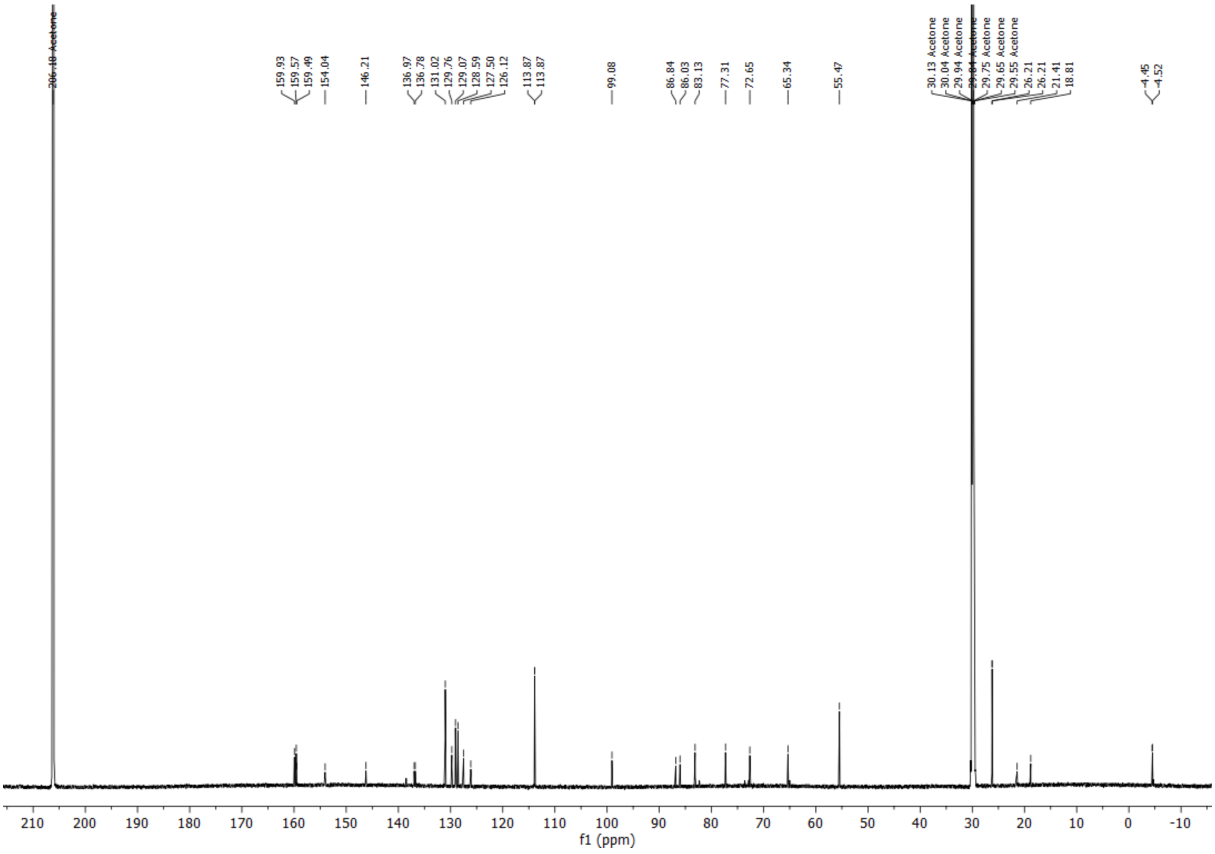

^{31}P {^{1}H} NMR spectra of compound α-8

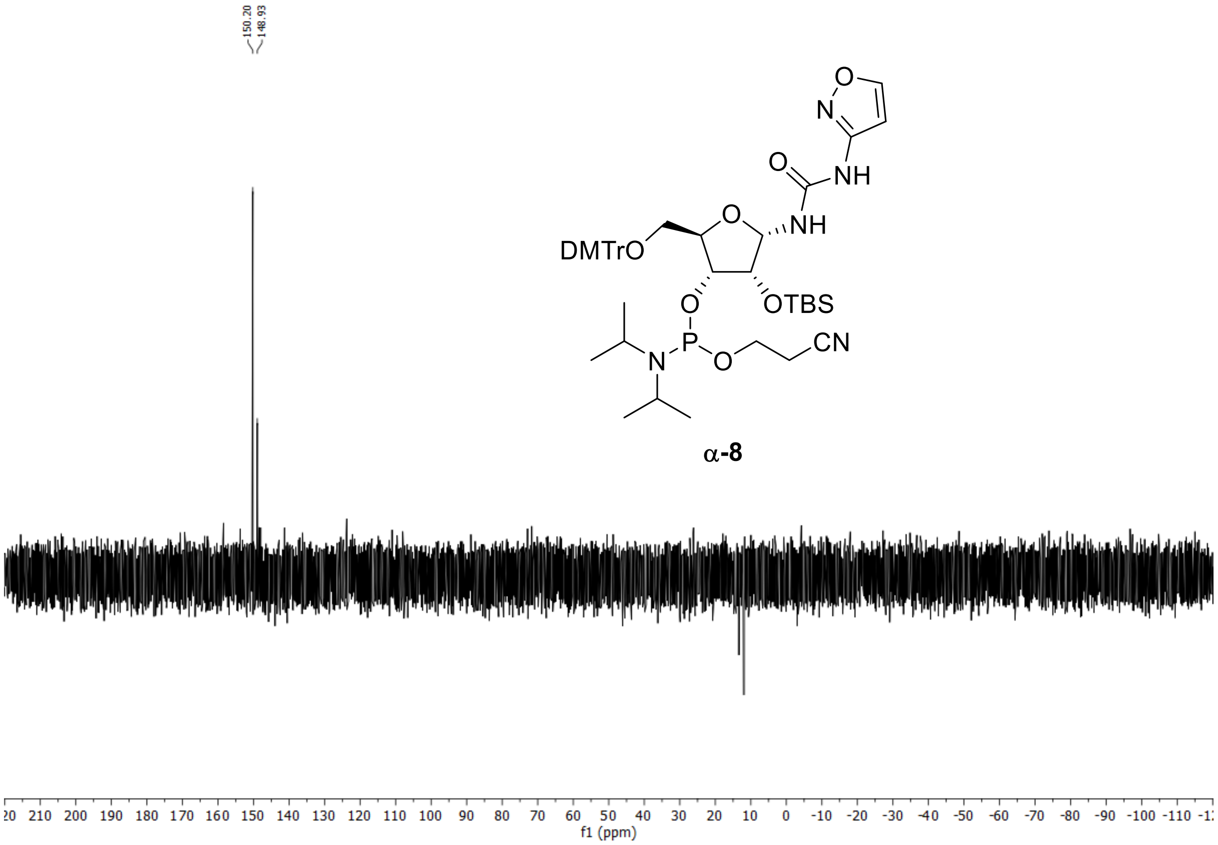

^{31}P {^{1}H} NMR spectra of compound β-8

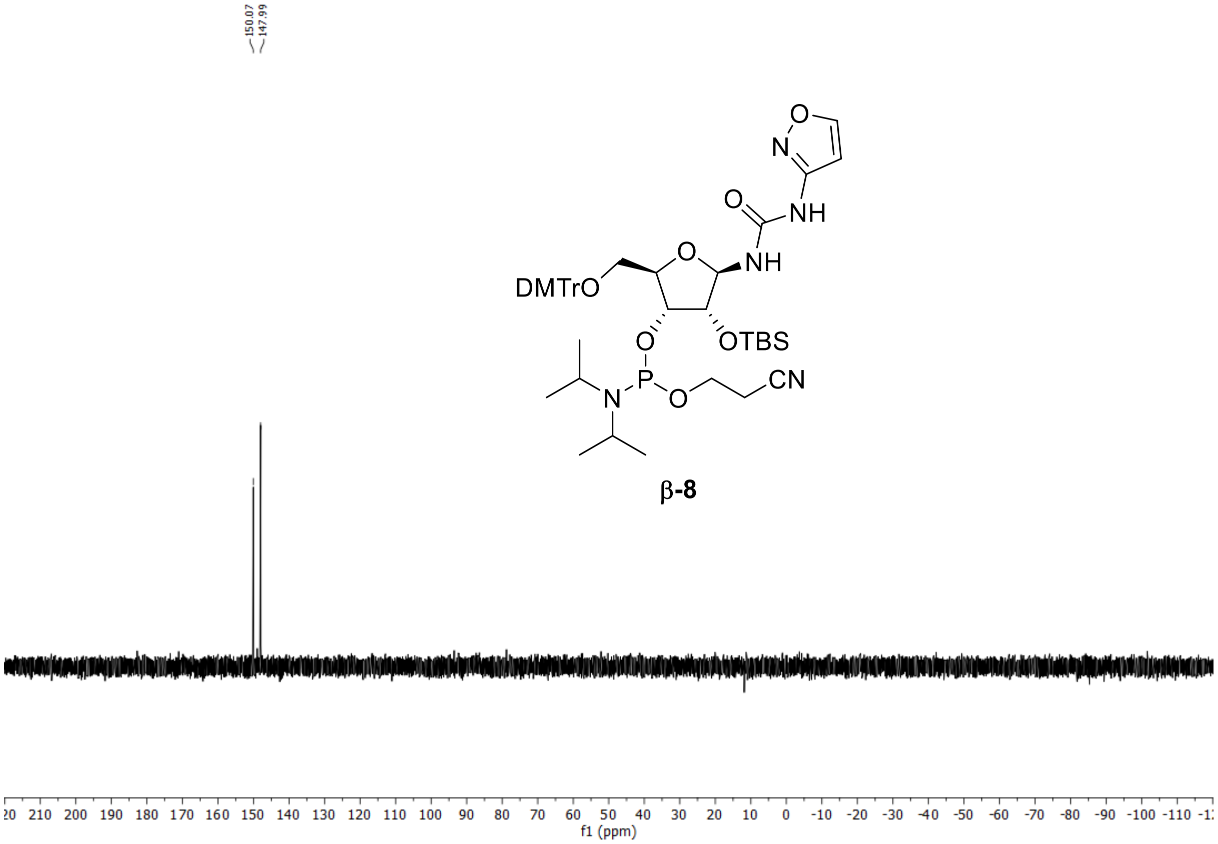

^{31}P {^{1}H} NMR spectra of compound β-8

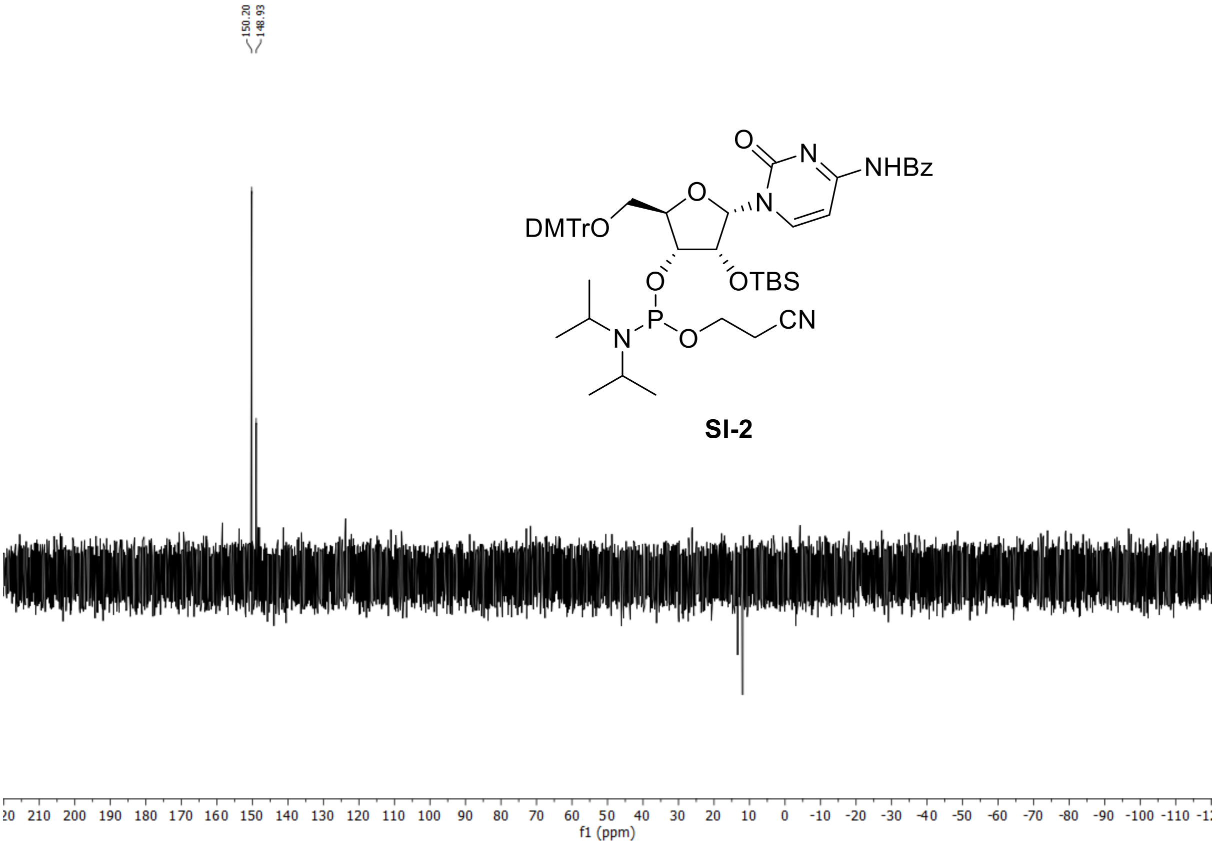

References

[1] H. Okamura, A. Crisp, S. Hübner, S. Becker, P. Rovó, T. Carell, *Angew. Chem. Int. Ed.* **2019**, *58*, 18691-18696.
[2] F. Debart, B. Rayner, G. Degols, J. L. Imbach, *Nucleic Acids Res.* **1992**, *20*, 1193-1200.
[3] S. Becker, J. Feldmann, S. Wiedemann, H. Okamura, C. Schneider, K. Iwan, A. Crisp, M. Rossa, T. Amatov, T. Carell, *Science* **2019**, *366*, 76.
[4] W. Lee, M. Tonelli, J. L. Markley, *Bioinformatics* **2015**, *31*, 1325-1327.
[5] A. T. Brunger, *Nature Protocols* **2007**, *2*, 2728-2733.
[6] M. P. Williamson, in *Modern Magnetic Resonance* (Ed.: G. A. Webb), Springer Netherlands, Dordrecht, **2006**, pp. 409-412.
